# Night Shift Work and Breast Cancer Incidence: Three Prospective Studies and Meta-analysis of Published Studies

**DOI:** 10.1093/jnci/djw169

**Published:** 2016-10-07

**Authors:** Ruth C. Travis, Angela Balkwill, Georgina K. Fensom, Paul N. Appleby, Gillian K. Reeves, Xiao-Si Wang, Andrew W. Roddam, Toral Gathani, Richard Peto, Jane Green, Timothy J. Key, Valerie Beral

**Affiliations:** **Affiliations of authors:** Cancer Epidemiology Unit, Nuffield Department of Population Health, University of Oxford, Oxford, UK (RCT, AB, GKF, PNA, GKR, XSW, AWR, TG, JG, TJK, VB); Worldwide Epidemiology, GlaxoSmithKline R&D, Uxbridge, UK (AWR); Department of Oncoplastic Breast Surgery, Oxford University Hospitals NHS Trust, Oxford, UK (TG); Clinical Trial Service Unit and Epidemiological Studies Unit (CTSU), Nuffield Department of Population Health, University of Oxford, Oxford, UK (RP)

## Abstract

**Background:** It has been proposed that night shift work could increase breast cancer incidence. A 2007 World Health Organization review concluded, mainly from animal evidence, that shift work involving circadian disruption is probably carcinogenic to humans. We therefore aimed to generate prospective epidemiological evidence on night shift work and breast cancer incidence.

**Methods:** Overall, 522 246 Million Women Study, 22 559 EPIC-Oxford, and 251 045 UK Biobank participants answered questions on shift work and were followed for incident cancer. Cox regression yielded multivariable-adjusted breast cancer incidence rate ratios (RRs) and 95% confidence intervals (CIs) for night shift work vs no night shift work, and likelihood ratio tests for interaction were used to assess heterogeneity. Our meta-analyses combined these and relative risks from the seven previously published prospective studies (1.4 million women in total), using inverse-variance weighted averages of the study-specific log RRs.

**Results:** In the Million Women Study, EPIC-Oxford, and UK Biobank, respectively, 673, 28, and 67 women who reported night shift work developed breast cancer, and the RRs for any vs no night shift work were 1.00 (95% CI = 0.92 to 1.08), 1.07 (95% CI = 0.71 to 1.62), and 0.78 (95% CI = 0.61 to 1.00). In the Million Women Study, the RR for 20 or more years of night shift work was 1.00 (95% CI = 0.81 to 1.23), with no statistically significant heterogeneity by sleep patterns or breast cancer risk factors. Our meta-analysis of all 10 prospective studies included 4660 breast cancers in women reporting night shift work; compared with other women, the combined relative risks were 0.99 (95% CI = 0.95 to 1.03) for any night shift work, 1.01 (95% CI = 0.93 to 1.10) for 20 or more years of night shift work, and 1.00 (95% CI = 0.87 to 1.14) for 30 or more years.

**Conclusions:** The totality of the prospective evidence shows that night shift work, including long-term shift work, has little or no effect on breast cancer incidence.

Light at night can suppress melatonin secretion, and it has been proposed that night shift work could increase breast cancer incidence (1). In a review of the evidence available in 2007, the World Health Organization’s International Agency for Research on Cancer (IARC) classified shift work that involves circadian disruption as a probable (breast) carcinogen, based on sufficient evidence from animal studies but only limited evidence of an effect on human breast cancer (2).

The available human evidence reviewed by IARC in 2007 was characterised as inadequate for assessing moderate risks reliably partly because of potential confounding and inconsistent definitions of shift work and partly because most studies were retrospective in design, comparing responses from women already diagnosed with breast cancer with those from unaffected women. Some retrospective results might have been moderately biased by differential recall, and/or by differential participation in the studies between women who had and had not worked night shifts (3–5). The only prospective information available in 2007 that directly compared breast cancer incidence in women reporting night shift work with that in other women was from two studies of US nurses, and elevated breast cancer rates were reported for 20 or more years of night shift work (6,7).

Since the IARC review, results from five further prospective studies have been published (2 in urban China and 3 in continental Europe) (8–12) and several meta-analyses have been done (13–18), although the most recent included results from only five of the prospective studies (18). To provide reliable epidemiological evidence, with minimum methodological bias, on any relationship between night shift work and breast cancer incidence, we now report first results from an additional three prospective studies and an updated meta-analysis of findings from all prospective studies, now 10 in total. 

## Methods

In response to the 2007 IARC review and a subsequent call from the Health and Safety Executive in the UK for further epidemiological research on breast cancer, we introduced questions on night shift work into our resurveys of two large UK prospective studies, and contributed to questions on night shift work in another large prospective study, UK Biobank. We also planned a meta-analysis of results from these studies with results from all other prospective studies.

### UK Study Participants, Data Collection, and Follow-Up

Participants from the Million Women Study (19), EPIC-Oxford (20), and UK Biobank (21,22) provided data on night shift work and other factors relevant for breast cancer risk. For each study, participants gave written consent. The design, methods, questionnaires, and ethics approvals for each study are on the study-specific websites (www.millionwomenstudy.org, www.epic-oxford.org, www.ukbiobank.ac.uk), and further details are given in the Supplementary Material (available online).

Questions on night shift work were inserted into resurvey questionnaires of Million Women Study and EPIC-Oxford participants to test the hypothesis that regular night shift work, particularly long-term night shift work, is associated with an increased incidence of breast cancer (23). Participants were asked whether they had ever regularly worked at night or on night shifts, using questions similar to those asked in previous prospective studies (6–8), and fewer than 3% in each study did not answer the night shift work question (Supplementary Methods, available online). Those who answered “yes” were asked about the duration, timing, and nature of their night shift work. The questionnaires were completed, on average, in 2011 by Million Women Study participants and in 2010 by EPIC-Oxford participants.

Recruitment for UK Biobank took place, on average, in 2008 (from 2006 to 2010). Participants were asked about their employment, and those employed were asked whether their current job involved night shifts never/rarely, sometimes, usually, or always.

Participants in the three prospective studies were followed via record linkage to the National Health Service (NHS) Central Registers, which provide information on cancer registrations and deaths, coded to the 10th revision of the International Classification of Diseases (ICD-10) (24). The endpoints in these analyses are first diagnosis of invasive breast cancer (ICD-10 C50) or death attributed to breast cancer (ICD-10 C50).

Women with any invasive cancer (except nonmelanoma skin cancer [ICD-10 C44]) or in situ breast cancer (ICD-10 D05) registered before the baseline shift work survey, and women with unknown night shift work status (ever/never or yes/no) were excluded from the analyses. Woman-years were calculated from the date women answered the night shift work questions to the date of cancer registration, death, or last follow-up (December 31, 2013 in the Million Women Study and EPIC-Oxford, and December 14, 2012 in UK Biobank), whichever was first.

### Meta-analysis and Identification of Prospective Studies

Relevant publications were identified from computer-aided literature searches and reviews up to December 31, 2015 (Supplementary Methods, available online). We searched MEDLINE/PubMed, Scopus, and Web of Science using combinations of the search terms “shift work,” “night work,” “breast cancer,” “cohort,” and “prospective.” Prospective studies are those where exposure data were recorded before the onset of breast cancer. Relative risks (RRs) and 95% confidence intervals (CIs) were extracted from prospective analyses for incident invasive breast cancer for any night shift work, and for 20 or more years of night shift work vs no night shift work (and, where possible, for ≥30 years).

### Statistical Analysis

In the three UK prospective studies, we categorized study participants into those who reported working or not working at night; ever or never having regularly worked night shifts for Million Women Study and EPIC-Oxford participants, and currently or not currently employed in a job involving night shift work for UK Biobank participants. Based on the hypothesis that longer duration of night shift work may be associated with a higher incidence of breast cancer, we also categorized workers by duration of night shift work: fewer than 10, 10 to 19, and 20 or more years in the Million Women Study and EPIC-Oxford (duration was not available for UK Biobank). Further analyses were done examining associations by other categories of duration of night shift work (<15, 15–29, and ≥30 years) and by time since last working night shifts (<10, ≥10 years before baseline).

Cox regression models were used to calculate breast cancer incidence rate ratios (RRs) and their 95% confidence intervals comparing various categories of women reporting night shift work with those who had never worked night shifts (the reference group).

The proportional hazards assumption was checked by inclusion in the model of an interaction term between duration of shiftwork and the underlying time variable (age). Since this term was not statistically significant, there was no evidence to suggest that the proportional hazards assumption was violated.

Attained age was the underlying time variable, and analyses were stratified by geographical region of recruitment (and for EPIC-Oxford additionally by method of recruitment via general practitioners or postal survey). Analyses were initially conducted without any further adjustment, and the main analyses with additional adjustment for: socioeconomic status, age at menarche, parity and age at first birth, body mass index, alcohol intake, smoking, strenuous physical activity, family history of breast cancer, living with a partner, use of oral contraceptives, and menopausal hormone therapy (categorization of the adjustment variables is described in the Supplementary Methods, available online). For each variable, missing values were assigned a separate category. Chi-squared tests for trend in RRs across night shift work duration categories involved inverse-variance weighted regression of the log RR in each category vs the mean duration within that category.

We also examined breast cancer incidence by night shift work in subgroups of Million Women Study participants defined by selected characteristics including diurnal preference (a graded response to preferring mornings or evenings, sometimes called chronotype), sleep patterns, adiposity, alcohol use, and history of working as a nurse for 10 or more years. To test for heterogeneity of the RR for night shift work across categories of these characteristics, we used likelihood ratio tests to compare multivariable Cox regression models with and without interaction terms for night shift work and the relevant factor.

For the meta-analyses, in studies where results for more than one category of duration of night shift work are available, a single study-specific estimate was calculated, taking into account the covariance between such estimates (25); this single study-specific RR was used in estimates of the summary RR in the meta-analysis. Summary RRs combining study-specific results were calculated from an inverse–variance weighted average of the study-specific log RRs. Unweighted chi-squared tests assessed heterogeneity across studies (calculation of a weighted average of several results does not require homogeneity between them).

All analyses used Stata version 14.0. Statistical tests were two-sided. Results with *P* values ≥ .05 were reported as not statistically significant, but interpretation of the *P* values took multiple testing into account.

## Results

The UK prospective studies of night shift work and breast cancer incidence included 522 246 postmenopausal women in the Million Women Study, 22 559 in EPIC-Oxford, and 251 045 in UK Biobank who had provided information on night shift work and were followed for incident breast cancer. In the Million Women Study and EPIC-Oxford, about one in seven reported ever having worked night shifts and about one in 50 reported working night shifts for 20 or more years (Table 1 and Supplementary Material, available online). In UK Biobank, 3.6% reported at baseline that their current job involved night shift work (Table 1). In the Million Women Study, a subset of participants (n = 1322) answered the questionnaire about shift work on two occasions, two months apart, with good agreement between the reports (Supplementary Material, available online).

**Table 1:** Baseline characteristics by reported night shift work in three UK prospective studies and results of follow-up for breast cancer

	Million Women Study*		EPIC-Oxford		UK Biobank	
	Never night shifts	Ever night shifts	Never night shifts	Ever night shifts	Not current night shifts	Current night shifts
Baseline characteristics and breast cancer follow-up	(n = 450 232)	(n = 72 014)	(n = 19 289)	(n = 3270)	(n = 241 972)	(n = 9073)
Baseline characteristics						
Mean age at baseline (SD), years	68.8 (4.7)	68.5 (4.6)	58.0 (12.2)	56.6 (11.7)	56.3 (8.0)	51.0 (6.6)
Socioeconomic status, No. (% in lower third)	145 392 (32.5)	26 129 (36.6)	5628 (33.0)	1009 (35.1)	79 450 (32.9)	4109 (45.4)
Not married nor living with a partner, No. (%)	66 529 (17.8)	14 006 (23.7)	5693 (29.6)	1093 (33.4)	72 399 (30.0)	3676 (40.7)
Nulliparous, No. (%)	49 313 (11.0)	7798 (10.8)	7216 (37.7)	1247 (38.5)	45 000 (18.7)	1922 (21.3)
Mean No. of children (parous women) (SD)	2.3 (0.9)	2.5 (1.0)	2.2 (0.9)	2.2 (1.0)	2.2 (0.9)	2.3 (1.0)
Mean age at first birth (parous women) (SD), years	24.3 (4.2)	24.0 (4.4)	26.1 (4.7)	26.0 (4.7)	26.0 (5.1)	25.3 (5.4)
Obese, No. (%)	63 554 (14.7)	13 307 (19.2)	1053 (5.6)	246 (7.7)	55 962 (23.6)	2670 (30.0)
Strenuous physical activity >2 h/wk, No. (%)	103 199 (23.4)	17 636 (25.1)	5512 (29.0)	1122 (34.6)	37 364 (17.5)	1717 (21.9)
Mean alcohol consumption (SD), g/d	6.4 (7.6)	6.1 (7.6)	8.1 (9.8)	7.7 (9.1)	8.9 (10.9)	8.5 (11.7)
Current smoker, No. (%)	57 198 (13.3)	13 122 (19.1)	1638 (8.5)	372 (11.4)	20 865 (8.7)	1390 (15.4)
First-degree relative with breast cancer, No. (%)	41 339 (9.7)	6783 (10.1)	N/A	N/A	15 522 (6.8)	471 (5.5)
Ever oral contraceptive user, No. (%)	281 855 (63.0)	48 044 (67.1)	14 798 (77.2)	2615 (80.4)	195 681 (81.1)	7671 (84.9)
Ever menopausal hormone therapy user, No. (%)†	226 674 (54.2)	41 679 (62.1)	1420 (40.2)	219 (45.9)	70 624 (51.4)	1361 (49.6)
Mean amount of sleep (SD), hours	6.8 (1.3)	6.7 (1.8)	6.9 (1.1)	6.8 (1.2)	7.2 (1.1)	7.0 (1.2)
Take medication to sleep on most days, No. (%)	22 613 (5.3)	4865 (7.0)	369 (1.9)	87 (2.7)	N/A	N/A
More evening than morning type, No. (%)	117 848 (28.8)	21 526 (33.1)	5252 (29.5)	1008 (33.8)	80 068 (36.4)	3683 (45.2)
Follow-up for breast cancer						
Mean person-years of follow-up per woman	2.6	2.6	3.1	3.1	3.8	3.9
Total No. of incident breast cancers	4136	673	153	28	2653	67

*For the Million Women Study, the tabulation is of strenuous physical activity more than once per week.

†Restricted to women aged 55 years or older.

Nursing was by far the most common job reported by female night shift workers. In the Million Women Study, 45.0% of all night shift workers and 61.1% of those reporting 20 or more years of night shift work had worked as a nurse for at least 10 years (Table 2). Among the female night shift workers in EPIC-Oxford, almost half reported having worked as a nurse, almost half had worked rotating night shifts, and night shift workers worked an average of 8.8 (SD = 5.9) nights per month and worked 10.2 (SD = 2.7) hours per night shift (Table 3).

**Table 2:** Specific jobs reported by the 72 014 night shift workers in the Million Women Study

Job description*	Ever night shift worker (n=72 014)	≥20 y of night shift work (n=9647)
Nurse, No. (%)	32 374 (45.0)	5899 (61.1)
Cleaner, No. (%)	4001 (5.6)	336 (3.5)
Factory worker, No. (%)	4438 (6.2)	310 (3.2)
Bar worker, No. (%)	1845 (2.6)	296 (3.1)
Shop worker, No. (%)	5012 (7.0)	288 (3.0)
Cook/waitress, No. (%)	2764 (3.8)	273 (2.8)
Flight attendant, No. (%)	368 (0.5)	102 (1.1)

* Women were asked about 15 specific jobs worked for at least 10 years. The jobs potentially associated with night shift work are shown in the table. The percentages are of the total in each night shift category.

**Table 3:** Characteristics of night shift work reported by 3270 night shift workers in EPIC-Oxford

	Ever night shifts	Total night shift work duration†		
		<10 y	10–20 y	≥20 y
Characteristics of night shift work*	(n = 3270)	(n = 1819)	(n = 705)	(n = 461)
Night shift workers with rotating shifts, No. (%)	1380 (48.4)	838 (51.3)	289 (46.2)	147 (38.1)
Night shift workers with permanent night shifts, No. (%)	621 (21.8)	367 (22.4)	122 (19.5)	87 (22.5)
Night shift workers with flexible/irregular night shifts, No. (%)	853 (29.9)	430 (26.3)	214 (34.2)	152 (39.4)
Night shift work for >5 nights/month, No. (%)	1928 (68.0)	1135 (69.4)	412 (66.9)	241 (62.6)
Mean night shifts/month (SD)	8.8 (5.9)	9.3 (6.1)	8.1 (5.2)	8.0 (5.4)
Mean hours per night shift (SD)	10.2 (2.7)	10.1 (2.6)	10.2 (2.9)	10.3 (3.1)
Mean age first worked night shifts (SD)	28.6 (10.2)	28.4 (10.7)	29.3 (9.5)	29.2 (9.1)
Mean total years of night shift work (SD)	9.5 (8.5)	4.1 (2.2)	12.8 (2.8)	26.0 (6.0)
Night shift workers who reported specific occupations, No.§	2982	1703	647	412
Nurses, No. (%)	1444 (48.4)	814 (47.8)	314 (48.5)	198 (48.1)
Other health care‡, No. (%)	438 (14.7)	193 (11.3)	113 (17.4)	102 (24.8)
Social care, No. (%)	334 (11.2)	218 (12.8)	60 (9.3)	30 (7.3)
Emergency services, No. (%)	116 (3.9)	53 (3.1)	36 (5.6)	21 (5.1)
Hospitality, No. (%)	108 (3.6)	59 (3.5)	25 (3.9)	15 (3.6)
Air/flight, No. (%)	76 (2.5)	46 (2.7)	14 (2.2)	13 (3.2)
Tele/radar/wireless, No. (%)	58 (1.9)	36 (2.1)	14 (2.2)	3 (0.7)
Retail, No. (%)	54 (1.8)	36 (2.1)	13 (2.0)	1 (0.2)
Other, No. (%)	354 (11.9)	248 (14.5)	59 (9.1)	29 (7.1)

*Values relate to the night job of longest duration, where known.

†Duration of night shift work was unknown for 285 night shift workers.

‡Other health care workers include medical doctors, midwives, and radiographers.

§The percentages below use these numbers as the denominator.

There were consistent differences in characteristics between women reporting night shift work and other women within each of the three UK prospective studies (Table 1; Supplementary Tables 1-3, available online). Women reporting night shift work were more likely to be of lower socioeconomic status, to be obese, to be current smokers, not to live with a partner, to take medications to help sleep, and to report preferring evenings rather than mornings (Table 1). Some of these differences were more pronounced when comparing women who had worked at night for 20 or more years to those who had never worked at night (Supplementary Tables 1 and 2, available online).

**Figure 1.**
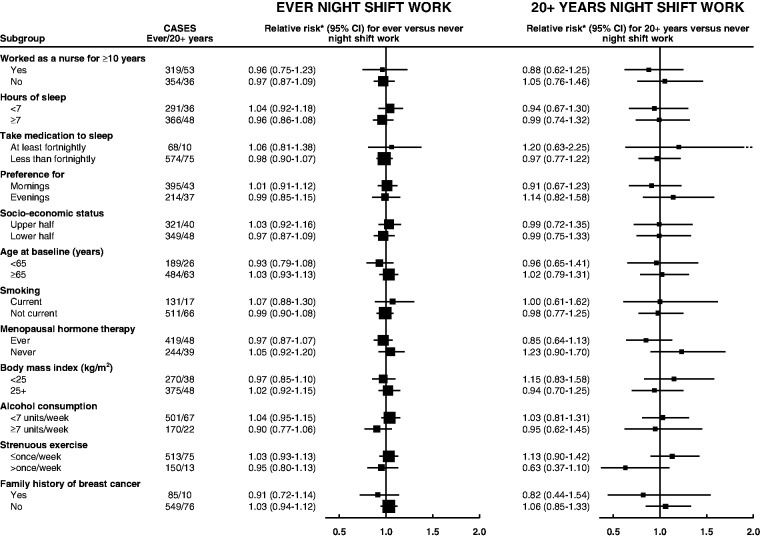
Relative risk of breast cancer in Million Women Study participants who worked night shifts by selected characteristics. *Relative to never night shift workers, stratified by region and with attained age as the underlying time variable and adjustment for socioeconomic status, parity and age at first birth, body mass index, alcohol intake, strenuous physical activity, family history of breast cancer, age at menarche, oral contraceptive use, smoking, living with a partner, and use of menopausal hormone therapy. Relative risks (RRs) are represented by **squares** (with their 95% confidence intervals [CIs] as **lines**), each with area inversely proportional to the variance of the log RR, thereby indicating the amount of statistical information for that particular RR.

In the Million Women Study, 4809 incident breast cancers were diagnosed during 1.4 million woman-years of follow-up after the night shift questionnaire. The adjusted breast cancer incidence rate ratio for ever vs never night shift work was 1.00 (95% CI = 0.92 to 1.08), with 673 cases among those who had reported night shift work (Table 4). Results were similarly null for night shift work durations of fewer than 10 years (RR = 0.93, 95% CI = 0.83 to 1.03), 10 to 19 years (RR = 1.14, 95% CI = 0.96 to 1.35), 20 or more years (RR = 1.00, 95% CI = 0.81 to 1.23), or 30 or more years (RR = 0.98, 95% CI = 0.69 to 1.39), and for night shift work within the last 10 years (RR = 1.10, 95% CI = 0.94 to 1.30) (Supplementary Table 4, available online). In analyses restricted to women who had been nurses for 10 or more years (Figure 1), the multivariable-adjusted RR of breast cancer for ever vs never night shift work was 0.96 (95% CI = 0.75 to 1.23); for 20 or more years of night shift work, it was 0.88 (95% CI = 0.62 to 1.25) (Supplementary Table 5, available online). In various other subgroups of women, there was no statistically significant heterogeneity across any of those considered (*P* > .05 for all tests) (Figure 1). For completeness, subgroup analyses are also shown in Figure 1 for 20 or more years of night shift work, but numbers are small. Among women for whom breast screening data were available, 56.1% and 55.9% of breast cancers were screen-detected in ever and never night workers, respectively, while 33.3% and 32.9% were interval cancers and 10.7% and 11.1% of breast cancers occurred in women who were eligible for screening but were not screened (all differences by screening status P > 0.05).

**Table 4:** Breast cancer incidence rate ratio by history of night shift work in 522 246 Million Women Study participants

	Breast cancer cases		Minimally adjusted	Multivariable-adjusted	
Night shift work	No.	Mean y of night shift work (SD)	RR*	RR† (95% CI)	
Ever worked at night					
Never	4136	–	1.00	1.00	
Ever	673	8.8 (8.9)	1.02	1.00 (0.92 to 1.08)	
Years of night shift work‡					
Never	4136	–	1.00	1.00	
<10	400	3.5 (2.2)	0.95	0.93 (0.83 to 1.03)	
10–19	140	12.5 (2.8)	1.18	1.14 (0.96 to 1.35)	
≥20	89	26.8 (7.1)	1.03	1.00 (0.81 to 1.23)	
*P*_trend_				.68^§^	

*Relative to never night shift workers, stratified by region and with attained age as the underlying time variable. CI = confidence interval; RR = incidence rate ratio from Cox regression models.

†Relative to never night shift workers, stratified by region and with attained age as the underlying time variable, and adjusted for socioeconomic status, parity and age at first birth, body mass index, alcohol intake, strenuous physical activity, family history of breast cancer, age at menarche, oral contraceptive use, smoking, living with a partner, and use of menopausal hormone therapy.

‡Duration of night shift work was unknown for 8184 night shift workers, among whom there were 44 cases of breast cancer.

§*P* is from a two-sided test for trend using mean years of night shift work within each duration category.

In EPIC-Oxford, 181 incident breast cancers were diagnosed during 70 000 woman-years of follow-up. Compared with women who had never worked night shifts, the multivariable-adjusted RR was 1.07 (95% CI = 0.71 to 1.62) for women who had ever worked night shifts (28 cases). There was only one case in an individual with 20 or more years of night shift work and no cases in individuals with 30 or more years of night shift work (Supplementary Table 6, available online).

In UK Biobank, 2720 incident breast cancers were diagnosed during 1.0 million woman-years of follow-up. Breast cancer incidence was not statistically significantly increased in women who were working night shifts at baseline; the multivariable-adjusted RR was 0.78 (95% CI = 0.61 to 1.00), based on 67 exposed cases. When women were categorized by their reported frequency of night shift work, the multivariable-adjusted RRs were 0.71 (95% CI = 0.50 to 1.00), 0.94 (95% CI = 0.54 to 1.67), and 0.85 (95% CI = 0.55 to 1.31) for women sometimes, usually, and always working night shifts, respectively, compared with women who never or rarely did so. No information was recorded about how long women had been doing night shifts. Restriction of analyses to women who had no missing data for any of the adjustment variables had little effect on the study-specific results (Supplementary Table 7, available online).

**Figure 2.**
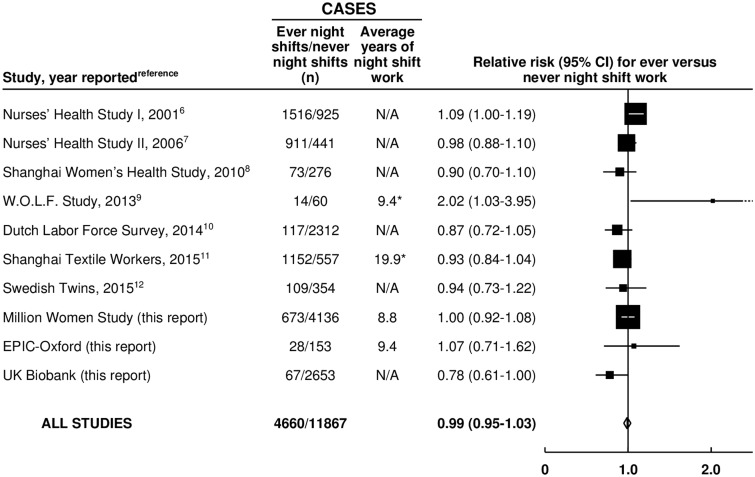
Meta-analysis of prospective studies on the risk of breast cancer in women who ever vs never worked night shifts. *All women. Study-specific relative risks (RRs) are represented by **squares** (with their 95% confidence intervals [CIs] as **lines**), each with area inversely proportional to the variance of the log RR. RRs were combined using inverse- variance-weighted averages of the log RRs in the separate studies, yielding a result and its 95% CI, which is plotted as a **diamond**.

Our systematic review identified 10 prospective studies (including the 3 UK studies reported here, total = 0.8 million women, and 7 other studies, total = 0.6 million women) that had reported on breast cancer incidence and shift work (Supplementary Table 8, available online) (6–12). Altogether, these studies included 4660 women with breast cancer who had worked night shifts (Figure 2). When results from the 10 studies were combined, the weighted average RR was 0.99 (95% CI = 0.95 to 1.03) for any night shift work compared with none. There was no statistically significant heterogeneity across studies (*P* = .052).

**Figure 3.**
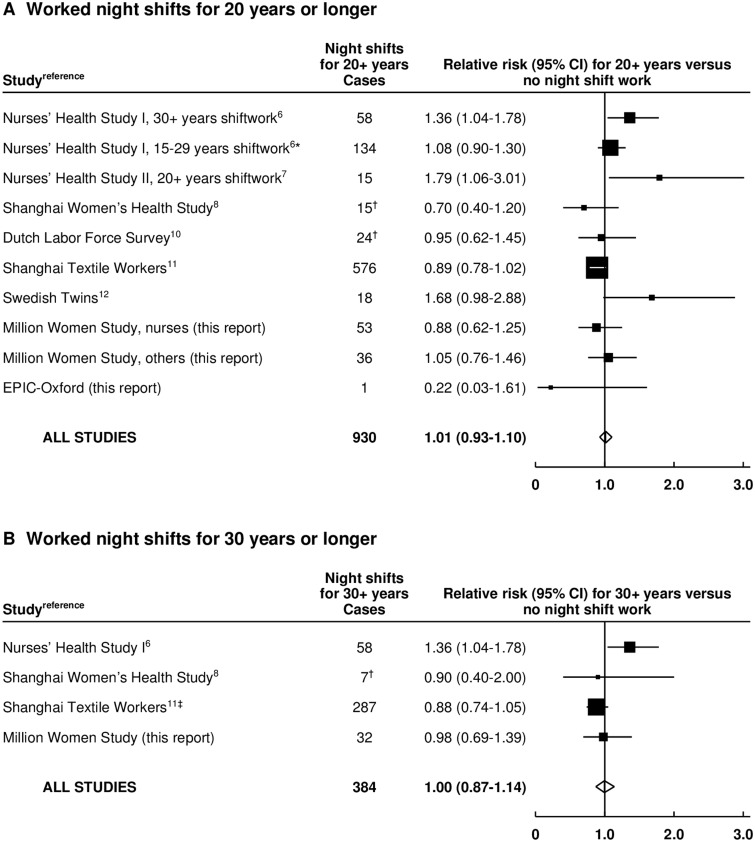
Meta-analysis of prospective studies on the risk of breast cancer associated with long-duration night shift work. **A)** Worked night shifts for 20 or more years vs never worked night shifts. **B)** Worked night shifts for 30 or more years vs never worked night shifts. *Results for 20 to 29 years not reported separately. †Approximate numbers, estimated from confidence limits. ‡Worked night shifts for more than 27.5 years. Study-specific relative risks (RRs) are represented by **squares** (with their 95% confidence intervals [CIs] as **lines**), each with area inversely proportional to the variance of the log RR. RRs were combined using inverse-variance-weighted averages of the log RRs in the separate studies, yielding a result and its 95% CI, which is plotted as a **diamond**.

Information on breast cancer incidence associated with 20 or more years of night shift work was available for eight of the 10 studies (Figure 3A) (6–8,10–12). One study of US nurses did not report the RR specifically for 20 or more years of night shift work, so we include its findings for 30 or more years and its findings for 15 to 29 years of night shift work (as in the 15–29 years category, most would have worked nights for ≥20 years) (6). For comparison with results from the Nurses’ Health Studies, the Million Women Study results are shown separately for nurses and other women.

Taking all eight studies together, the combined RR associated with 20 or more years of night shift work was 1.01 (95% CI = 0.93 to 1.10) (Figure 3A), with no strong heterogeneity across studies (*P* = 0.011). These meta-analyses were repeated using updated results from the Nurses’ Health studies, which have been published in a conference abstract only (26) (for Nurses' Health Study I, RR for ≥30 years versus never = 0.95, 95% CI = 0.77–1.17; and for Nurses' Health Study II, RR for ≥20 years versus never = 1.33, 95% CI = 0.93–1.89). The combined relative risk for 20 or more years was 0.97 (95% CI=0.90 to 1.06) with no significant heterogeneity (P=0.12) (Supplementary Table 8 and Supplementary Figure 1, available online).

Information on night shift work for 30 or more years was available for only four studies (6,8,11). This incompleteness limits what can be concluded about such prolonged night shift work, but the combined RR was 1.00 (95% CI=0.87 to 1.14, P heterogeneity = 0.067)(Figure 3B).

## Discussion

The totality of the prospective evidence, from three new UK prospective studies and seven other prospective studies, shows no evidence of any association of breast cancer incidence with night shift work and, in particular, no evidence of any increase in incidence with 20 or more years of night shift work. Confidence intervals for the incidence rate ratios are narrow, even for 20 or more years of night shift work (RR = 1.01, 95% CI = 0.93 to 1.10), so these findings exclude a moderate association of breast cancer incidence with long duration night shift work.

The main limitation of the present findings is that an increase in the relative risk of breast cancer incidence of only a few percent cannot be ruled out. This is partly because the total number of cases of breast cancer arising in women in prospective studies who had reported long-term night shift work is still less than 1000; this will increase with longer follow-up and publication of further studies, but is already more than four times as many as the number available for the 2007 IARC review (2). It is also partly because women who have worked night shifts differ in several respects from women who have not (23), so residual confounding cannot be completely excluded. In the three UK studies, women who had worked night shifts were somewhat more likely to be obese, to smoke, to take medications to help them sleep, and to prefer evenings rather than mornings. Nevertheless, we observed no association of night shift work with breast cancer incidence in any of these UK studies, either in minimally adjusted or in multivariable-adjusted analyses. In UK Biobank, information was available only for current shift work. Omission of this study from the totality of the evidence would make little difference to the results for ever vs never night shift work and no difference to the results for 20 or more years of night shift work.

It had been hypothesised that any adverse associations of night shift work with breast cancer incidence may be masked by differences in breast screening in night shift workers, but in the Million Women Study we found no differences by night shift work category in the proportion of breast cancers detected by screening. We also found no evidence that the null association between night shift work and breast cancer incidence was modified by personal characteristics such as sleep characteristics (including diurnal preference), family history of breast cancer, or recency of night shift work. 

The conclusions of this report are strengthened by the limited heterogeneity between study-specific results, despite differences in design, population studied, exposure definition and assessment, night shift pattern, and control of potential confounders. Notably, while some studies focused on one occupation or industry, eg, nurses (6,7) or textile workers (11), others included participants from many occupations (9) or the general population (8,10,12). To test whether exposures specific to night shift work among nurses rather than in other occupations could be relevant (6,7), sensitivity analyses of Million Women Study participants who had worked for 10 or more years as a nurse were done, but they showed no statistically significant evidence of increased breast cancer incidence with long-term night shift work.

Individual-level night shift exposure information was self-reported in these three studies and five others (6–9, 12) and obtained via linkage to a workforce survey in one study (10). In the 10th study, it was assessed by combining individual-level information on employment in specific manufacturing processes within a particular factory with data on night shift work associated with each specific process in that factory (11). Repeatability of self-reported night shift work in the Million Women Study was good for reporting of any night shift work and the duration of night shift work. Women who reported long-duration night shift work are likely to have had substantial exposure to night shift work, so misclassification is unlikely to have been so great as to have masked any material risk.

The 10 studies in this meta-analysis all had data on night shift work that had been collected prospectively and included a total of 1.4 million women. Five of these prospective studies, which included a total of 0.8 million women, have been published in the last two years, and their results were not included in the most recent meta-analyses (14–18). Restriction to prospective studies is important when trying to detect or refute moderate hazards as it avoids the moderate biases that can result from retrospective methodology. The totality of the current prospective evidence suggests that night shift work, including long-term night shift work, has little or no effect on breast cancer incidence. The IARC 2007 shift work review was necessarily based on limited epidemiological evidence, and, although further follow-up is desirable, the prospective evidence now available shows that classification of night shift work as a probable human (breast) carcinogen is no longer justified.

### Funding

This work was supported by the UK Health and Safety Executive (contract number JN2995), Cancer Research UK (grant numbers C570/A16491 and C8221/A19170), and the Medical Research Council (grant numbers MR/K02700X/1 and MR/M012190/1). This research uses the UK Biobank Resource. 

### Notes

The sponsors had no role in the design of the study; the collection, analysis, or interpretation of the data; the writing of the manuscript; or the decision to submit the manuscript for publication. The authors had full access to the data and analyses and were solely responsible for the decision to submit for publication. All authors contributed to the design and execution of this work, and to the preparation of the report. All authors had an opportunity to contribute to the interpretation of the results and to redrafting, and all approved the final report.

We declare that we have no conflicts of interest.

Million Women Study Collaborators**:** The Million Women Study Advisory Committee: Emily Banks, Valerie Beral, Lucy Carpenter, Carol Dezateux, Jane Green, Julietta Patnick, Richard Peto, Cathie Sudlow. National Health Service (NHS) Breast Screening Centres that took part in the recruitment and breast screening follow-up for the Million Women Study (in alphabetical order): Avon, Aylesbury, Barnsley, Basingstoke, Bedfordshire and Hertfordshire, Cambridge and Huntingdon, Chelmsford and Colchester, Chester, Cornwall, Crewe, Cumbria, Doncaster, Dorset, East Berkshire, East Cheshire, East Devon, East of Scotland, East Suffolk, East Sussex, Gateshead, Gloucestershire, Great Yarmouth, Hereford and Worcester, Kent, Kings Lynn, Leicestershire, Liverpool, Manchester, Milton Keynes, Newcastle, North Birmingham, North East Scotland, North Lancashire, North Middlesex, North Nottingham, North of Scotland, North Tees, North Yorkshire, Nottingham, Oxford, Portsmouth, Rotherham, Sheffield, Shropshire, Somerset, South Birmingham, South East Scotland, South East Staffordshire, South Derbyshire, South Essex, South Lancashire, South West Scotland, Surrey, Warrington Halton St Helens and Knowsley, Warwickshire Solihull and Coventry, West Berkshire, West Devon, West London, West Suffolk, West Sussex, Wiltshire, Winchester, Wirral, and Wycombe. The Million Women Study Co-ordinating Centre staff: Hayley Abbiss, Simon Abbott, Rupert Alison, Naomi Allen, Miranda Armstrong, Krys Baker, Angela Balkwill, Isobel Barnes, Valerie Beral, Judith Black, Roger Blanks, Kathryn Bradbury, Anna Brown, Benjamin Cairns, Dexter Canoy, Andrew Chadwick, Barbara Crossley, Dave Ewart, Sarah Ewart, Lee Fletcher, Sarah Floud, Toral Gathani, Laura Gerrard, Adrian Goodill, Jane Green, Lynden Guiver, Michal Hozak, Isobel Lingard, Sau Wan Kan, Nicky Langston, Kath Moser, Kirstin Pirie, Gillian Reeves, Keith Shaw, Emma Sherman, Helena Strange, Sian Sweetland, Sarah Tipper, Ruth Travis, Lyndsey Trickett, Lucy Wright, Owen Yang, and Heather Young.

We thank Adrian Goodill for the preparation of the figures.

## Supplementary Material

Supplementary Data

Supplementary Data
