# Supplementary material for: Night Shift Work and Breast Cancer Incidence: Three Prospective Studies and Meta-analysis of Published Studies
Source: J Natl Cancer Inst. 2016 Oct 7;108(12):djw169. doi: 10.1093/jnci/djw169 (PMC5241898; doi:10.1093/jnci/djw169)
Supplement: Supplementary Data [file supp_108_12_djw169__index.html]

Night Shift Work and Breast Cancer Incidence: Three Prospective Studies and Meta-analysis of Published Studies — Supplementary Data 

# Night Shift Work and Breast Cancer Incidence: Three Prospective Studies and Meta-analysis of Published Studies

## Supplementary Data

files

- Supplementary Data - docx file
