# Supplementary material for: Night Shift Work and Breast Cancer Incidence: Three Prospective Studies and Meta-analysis of Published Studies
Source: J Natl Cancer Inst. 2016 Oct 7;108(12):djw169. doi: 10.1093/jnci/djw169 (PMC5241898; doi:10.1093/jnci/djw169)
Supplement: Supplementary Data [file supp_djw169_CLEAN_unlinked_RT_16-0223R1_Travis_supp_mat_31_8_2016.docx]

**Night shift work and breast cancer incidence: three prospective studies and meta-analysis of published studies**

Ruth C Travis DPhil, Angela Balkwill MSc, Georgina K Fensom MSc, Paul N Appleby MSc, Prof Gillian K Reeves PhD, Xiao-Si Wang PhD, Andrew W Roddam PhD, Toral Gathani MD, Prof Richard Peto FRS, Prof Jane Green DPhil, Prof Timothy J Key DPhil, Prof Valerie Beral FRS

**Supplementary Material**

**SUPPLEMENTARY METHODS**

**The Million Women Study**

During 1996-2001, a total of 1.3 million women aged 50-64 years who had been invited for routine screening for breast cancer at 66 screening centres in England and Scotland completed the recruitment questionnaire. The questionnaire asked about various characteristics, including anthropometric and reproductive factors, and other personal characteristics. Since recruitment, four questionnaires have been sent to the cohort participants approximately every 3-4 years to update and expand information on various factors. The study design, methods, survey questionnaires and information about data-sharing can be found at the study’s website (www.millionwomenstudy.org). The Oxford and Anglia Multi-Centre Research Ethics Committee and the Eastern Multi-Centre Research and Ethics Committee granted ethics approval for the work.

The fourth survey questionnaire was sent out in batches to the study population in 2009-12, and included questions for the first time about women’s history of night shift work, as described previously (1). There is not a universal definition of shift work and a variety of definitions have been used in previous shift work studies, depending on the main hypothesis of interest, study design, and study population. The main aim of the Million Women Study shift work project is to assess the hypothesis that regular night shift work, particularly long-term night shift work, is associated with increased risk for breast cancer and cardiovascular disease. Participants were therefore asked “Have you ever regularly worked at night or on night shifts (at any time between midnight and 06:00 hours, for at least 3 nights per month)?”. Those who answered “yes” were asked about the duration (“Over how many years in total?”) and timing of night shift work (“When did you last work at night?”). Previous large prospective studies of night shift work and breast cancer have also assessed duration of regular night shift work, with regular night shift work being similarly defined as night shift work undertaken ≥3 nights per month (2-4). The fourth survey questionnaire also asked about the women’s current use of hormone therapy for the menopause (HRT), diurnal preference (also sometimes referred to as chronotype; whether a woman considers herself to be a morning person, more of a morning than evening person, more of an evening than morning person or more of an evening person), and whether they had worked in certain specified jobs for ≥10 years.

In a subset of participants who completed a second shift work survey on average two months after the first baseline shift work survey (n=1322), responses from the baseline and repeat survey were compared using Cohen’s kappa statistic for agreement (5) and Spearman’s rank correlation coefficient. Repeat self-reports of night shift work history, including night shift work duration, showed good agreement with baseline data: there was 95.8% agreement between the responses in the first and repeat surveys to the question ‘Have you ever worked regularly at night, or on night shifts?’ (Cohen’s kappa coefficient = 0.86, Spearman’s rank correlation coefficient = 0.86). For the classification of women who had worked night shifts into night shift work duration categories (<10, 10-19 and ≥20 years), there was 87.0% agreement between the first and repeat surveys (Cohen’s kappa coefficient = 0.76, Spearman’s rank correlation coefficient = 0.77).

Participants from the Million Women Study are flagged on the NHS Central Registers so that cancer registrations and deaths can be routinely notified to the investigators. The Central Registers provide information on the date of each event and code the cancer site and cause of death according to the 10^th^ revision of the International Classification of Diseases (ICD) (6). The endpoints included in these analyses are first diagnosis of invasive breast cancer (ICD-10 C50) and death attributed to breast cancer (ICD-10 C50). Women with any invasive cancer (except non-melanoma skin cancer [ICD-10 C44]) or in situ breast cancer (ICD-10 D05) registered before the baseline shift work survey, and women with unknown night shift work status (ever/never) (n=9560, 1.8%) were excluded from the analyses. Baseline for these analyses is the date of fourth survey when information on night shift work was recorded. Woman-years were calculated from the date of fourth survey to the date of cancer registration (diagnosis of first malignant neoplasm, except non-melanoma skin cancer, or diagnosis of in situ breast cancer), death, loss to follow-up or the last date of follow-up (December 31, 2013), whichever was first.

The current analyses include data from 522 246 Million Women Study participants. Due to missing information on night shift work duration (which was unknown for 3184 women who had worked night shifts) and time since last worked night shifts (which was unknown for 4061 women who had worked night shifts), analyses according to night shift work duration include 519 062 women and analyses by time since last worked night shifts include 518 185 women.

*Statistical analysis*

We categorized the study participants into those who had ever and worked at night, and based on the hypothesis that longer duration of night shift work may be associated with a higher risk of breast cancer, women who had worked night shifts were also categorised into three groups according to their total duration of night shift work: short- (<10 years), medium- (10-19 years) and long-duration night shift workers (≥20 years).

Crude distributions of various characteristics by night shift work history were calculated. Cox regression models were used to obtain hazard ratios (hereafter referred to as incidence rate ratios [RRs]) and their 95% confidence intervals (95% CIs) for incident breast cancer comparing (i) women who had worked night shifts with other women (the reference group), and (ii) three categories of night shift workers defined according to total night shift work duration versus non-night workers (the reference group). Attained age was the underlying time variable and analyses were stratified by geographical region of recruitment (ten UK cancer registry regions). Analyses were also additionally adjusted for the following characteristics (from the recruitment survey unless otherwise stated): quintiles of socioeconomic status (based on Townsend deprivation index for area of residence at recruitment) (7); age at menarche (<12, 12-13, 14 or more years), parity and age at first birth (nulliparous, 1-2, or 3 or more births cross-classified by age at first birth <25, ≥25 years), body mass index (<25, 25-30, ≥30 kg/m^2^), alcohol intake (0, 1-2, 3-6, 7-14, ≥15 drinks/week), smoking (never, past, current <15 cigarettes/day, current ≥15 cigarettes/day), strenuous physical activity (never/rarely, less than weekly, at least weekly), family history of breast cancer (yes, no), married/living with a partner (yes, no), use of oral contraceptives (never, ever), and HRT use (never, past, current). For each adjustment variable, missing values were assigned to a separate category. Tests for linear trend in risk across night shift work duration categories were obtained by scoring the night shift work duration categories with values equal to the mean duration within that night shift work duration category.

**EPIC-Oxford**

EPIC Oxford is a prospective cohort study of 63 429 women and men aged ≥20 years who were recruited from around the United Kingdom between 1993 and 1999. A detailed description of the study and recruitment process has been published previously (8). Briefly, the study was designed primarily to investigate diet, lifestyle and risk of cancer among individuals with different dietary habits and therefore aimed to recruit vegetarians and vegans as well as participants from the general population. Recruitment was through general practice surgeries and through postal recruitment, and participants completed a questionnaire, which asked about diet, anthropometry, lifestyle and reproductive factors, prior disease and other personal characteristics. Subsequently, follow-up questionnaires have been sent to the cohort participants approximately every 5 years to update and expand information on various factors. The study design, methods, survey questionnaires and information about data sharing can be found at the study’s website ([www.epic-oxford.org](http://www.epic-oxford.org)). The protocol for the EPIC-Oxford study was approved by the multi-centre research ethics committee now called ‘Scotland A Research Ethics Committee’, and all participants gave written informed consent.

Participants from the EPIC-Oxford cohort were followed-up via record linkage to the NHS Central Registers, which provide information on cancer registrations and deaths, until the censoring date of December 31, 2013. The endpoints included in these analyses are first diagnosis of invasive breast cancer (ICD-10 C50) and death attributed to breast cancer (ICD-10 C50). Baseline for these analyses is the date of fourth survey when information on night shift work was recorded. Women were not eligible for this study if they had a registration for any malignant neoplasm (except non-melanoma skin cancer [ICD-10 C44]) or for in situ breast cancer (ICD-10 D05) with the NHS Central Register prior to the fourth survey, were aged 90 or over at recruitment, or did not provide information on night shift work (ever/never) (n=683, 2.9%). Woman-years were calculated from the date of fourth survey to the date of cancer registration (first malignant neoplasm, except non-melanoma skin cancer [ICD-10 C44], or in situ breast cancer [ICD-10 D05]), death, emigration or loss to follow-up, the participant’s 90^th^ birthday, or the censoring date, whichever occurred first.

The fourth questionnaire was sent out in 2010 and included questions for the first time about individual’s history of night shift work. The night shift work definition and questions were based on the main questions and definitions used in previous large prospective studies as described above (1-3), and reflect the main aim of the Oxford-based shift work and disease study, which is to assess the hypothesis that long-term night shift work is associated with increased incidence of breast cancer and cardiovascular disease. Participants were therefore asked “Have you regularly worked at night, on night shifts or on call at night? (Please only consider any job lasting for at least one year, and occurring on a regular basis for at least one night per month or 12 nights per year)”. Those who answered “yes” were asked about the overall duration (“Over how many years in total?”) and timing of the night shift work (“When did you last work nights?”), before being asked to provide more detailed information about each job which had involved night shift work (“How many different jobs have you had involving night work?”) and for each were asked to provide information on nights per month, hours per night and shift pattern [fixed/permanent, rotating, flexible/irregular].

Ever night shift workers were also asked about the occupation of their night shift work, providing a free-text response to the question ‘Please write down the occupation’. To categorise the free text response for the longest night work job worked by each night shift workers, the Stata command ‘strpos’ was used to search for a specific word or string of letters. Each category was manually checked to ensure that jobs had been categorised correctly, and the different categories were cross-tabulated against each other to check for overlap. The uncategorized jobs were put into a separate category and these were then manually checked and assigned to an appropriate category. Due to small numbers in certain occupation categories, several categories were merged as follows: ‘other health care’ includes doctors, midwives, radiographers and other health care workers’, ‘emergency services’ includes police and paramedics, and ‘other’ comprises reception/communication, computing, manufacturing, forces, royal mail, vets, media/publishing, transport, volunteers/helpline and all participants whose self-reported occupation for their night shift job did not fit into another occupation category.

In a subset of participants who completed a second shift work survey (on average four months) after the first baseline shift work survey (n=84), repeat self-reports of night shift work history and night shift work duration showed good agreement with baseline data. There was 97.5% agreement between the responses in the first and repeat surveys to the question ‘Have you ever worked regularly at night, or on night shifts?’ (Cohen’s kappa coefficient = 0.90, Spearman’s rank correlation coefficient = 0.91). For the classification of night shift workers into night shift work duration categories (<10, 10<20 and ≥20 years), there was 96.2% agreement between the first and repeat surveys (Cohen’s kappa coefficient = 0.84, Spearman’s rank correlation coefficient = 0.84).

The current analyses include data from the 22 559 EPIC-Oxford female participants who provided information on their night shift work history on the fourth survey. Duration of night shift work was unknown for 285 night shift workers, thus analyses by night shift work duration are based on data from 22 274 women.

*Statistical analysis*

Women were grouped according to their history of night shift work, firstly being categorised according to whether they had ever or never worked at night (with ever night shift work being defined as night shift work for at least one year), and secondly grouping those who had worked at night by their duration of night shift work (short <10 years, medium 10-19 years and long-duration ≥20 years) based on the hypothesis that a longer duration of night shift work might be associated with a higher risk of breast cancer.

Crude distributions of various characteristics by night shift work history were calculated. Cox regression models were used to obtain RRs and their 95% confidence intervals (95% CI) for incident breast cancer comparing (i) night shift workers versus non-night workers (the reference group), and (ii) three categories of night shift workers defined according to total night shift work duration versus non-night workers (the reference group). Attained age was the underlying time variable and analyses were stratified by geographical region of recruitment (twelve UK cancer registry regions) and by method of recruitment (postal or general practice surgeries). Analyses were additionally adjusted for the following characteristics (from the recruitment survey unless otherwise stated): quintiles of socioeconomic status (based on Townsend deprivation index for area of residence) (7); age at menarche (<12, 12-13, ≥14 years), parity and age at first birth (nulliparous, or 1-2, ≥3 births cross-classified by age at first child <25, ≥25 years), body mass index (<20, 20<22.5, 22.5<25, 25<27.5, ≥27.5 kg/m^2^), alcohol intake (<1, 1-7, 8-15, ≥16 g/day), smoking (from the fourth survey; never, past, current <15 cigarettes/day, current ≥15 cigarettes/day), strenuous physical activity (never, <2 hours per week, ≥2 hours per week), married or living as married (yes, no), use of oral contraceptives (never, ever) and HRT use (never, ever). For each adjustment variable, missing values were assigned to a separate category.

**UK Biobank**

UK Biobank is a prospective cohort study of 503 325 people, including 270 000 women, who were enrolled during 2006-2010 at 22 assessment centres across England, Wales and Scotland using standardised procedures (details of the UK Biobank protocol and data access are available at <http://www.ukbiobank.ac.uk/wp-content/uploads/2011/11/UK-Biobank-Protocol.pdf>). It was designed to be a resource for research into the causes of disease in middle and old age. Detailed descriptions of the study and recruitment process have been published previously (9,10). All individuals aged 40-69 who were registered with the National Health Service and living up to within ~25 miles of one of the assessment centres were invited to participate through postal invitation with a telephone follow-up, and the overall response rate from the ~9.2 million invitations was 5.47%. During an assessment centre visit, participants completed a wide ranging assessment that included a computerized touch-screen questionnaire on lifestyle, behavioural, dietary and socioeconomic factors, including employment, and on medical history, a face-to-face interview, and physical measurements, including body mass index assessment. Detailed information about the assessment procedure is available at <http://www.ukbiobank.ac.uk/>. The UK Biobank study was approved by the North West Multi-Centre Research Ethics Committee and all participants provided written informed consent to participate in the UK Biobank study.

Participants who indicated on the touch screen questionnaire that they were in paid employment or were self-employed, except those who indicated their work never or rarely involved shift work, were asked “Does your work involve night shifts?”, with possible answers being “Never/rarely”, “Sometimes”, “Usually”, “Always”, “Don’t know” and “Prefer not to answer”. A help button was available on the touchscreen and if a participant activated the help button they were shown the message “If you have more than one 'current job' then answer this question for your MAIN job only. Night shifts are a work schedule that involves working through the normal sleeping hours, for instance working through the hours from 12am to 6am.”

Women were not eligible for this study if they had a registration for any malignant neoplasm (except non-melanoma skin cancer [ICD-10 C44]) (n=16531) or for in situ breast cancer (ICD-10 D05) with the NHS Central Register at recruitment (n=4288), or did not provide information on current employment or night shift work (n=1602, of whom 1430 women did not provide information on employment status and so were not asked about night shift work, and 172 provided information on employment status but not on night shift work). The current analyses include data from the 251 045 UK Biobank participants.

*Flagging and follow-up*

Participants from UK Biobank were followed-up via record linkage to the NHS Central Registers, which provide information on cancer registrations and deaths, until the censoring date of December 14, 2012. The endpoints included in these analyses are first diagnosis of invasive breast cancer (ICD-10 C50) and death attributed to breast cancer (ICD-10 C50). Woman-years were calculated from the date of assessment centre visit to the date of cancer registration (first malignant neoplasm, except non-melanoma skin cancer [ICD-10 C44], or in situ breast cancer [ICD-10 D05]), death, loss to follow-up, or the censoring date, whichever occurred first.

*Statistical analysis*

Women were grouped according to employment, firstly being categorised according to whether their work involves any night shifts (no if answering “never/rarely” and yes if answering “sometimes”, “usually” or “always”), and secondly categorising women with work involving night shifts into three groups by their reported night shift frequency (sometimes, usually and always). Crude distributions of various characteristics by night shift work were calculated. Cox regression models were used to obtain RRs and their 95% confidence intervals (95% CI) for incident breast cancer comparing (i) night shift workers versus non-night (the reference group) and (ii) three categories of night shift workers defined according to frequency of night shift work versus non-night workers (the reference group). Attained age was the underlying time variable and analyses were stratified by geographical region of recruitment (ten UK regions). Analyses were additionally adjusted for quintiles of socioeconomic status (based on Townsend deprivation index for area of residence at recruitment)(7); age at menarche (<12, 12-13, ≥14 years), parity and age at first birth (nulliparous, 1-2, or ≥3 births cross-classified by age at first child <25, ≥25 years), body mass index (<25, 25-30, ≥30 kg/m^2^), alcohol intake (<1, 1-7, 8-15, ≥16 g/day), smoking (never, past, current <15 cigarettes per day, current ≥15 cigarettes per day), vigorous physical activity (never/rarely, up to 1 hour per week, >1 hour per week), family history of breast cancer (yes, no), living with a partner (yes, no), use of oral contraceptives (never, ever), and HRT use (never, past use at baseline, current use at baseline). For each adjustment variable, missing values were assigned to a separate category.

**Meta-analysis**

Previous meta-analyses of night shift work and breast cancer risk (11-17) have used heterogeneous eligibility criteria in terms of the exposure definitions, study design and eligibility period (see **Supplementary Table 8**). The most recent meta-analysis included findings from prospective studies published before October 2014 (17).

To put the results of the present study into the context of previous research, we conducted an updated meta-analysis combining our results with those from previously published prospective studies of the association between night shift work, including long duration night shift work, and breast cancer risk. We followed standard criteria for meta-analyses (MOOSE guidelines) (18). Relevant publications were identified from reviews and computer-aided literature searches (using MEDLINE/PubMed, Scopus, and Web of Science, with the keywords breast cancer [incidence or mortality], night work, shift work, cohort and prospective) independently by two researchers up to June 30, 2015. Studies were eligible for these meta-analyses if they included prospectively collected individual level data on work history and on other relevant risk factors for breast cancer, or had equivalent information for both women who had worked night shifts and non-night workers. One cohort study, a register-based retrospective cohort study based on record linkage to information from population censuses on occupation, was not eligible because of the study design; limited information was available on night shift work exposure (night shift workers were defined as individuals working in job titles and industry combinations [from the censuses] with at least 40% shift work and the reference group were people in occupation–industry combinations in which less than 30% stated that they were shift workers) and no information was available on established risk factors for breast cancer (19). Six nested case-control studies were not eligible because of study design; individual level exposure information on night work was not available (20-22) or data were collected retrospectively from study participants (by interview or questionnaire) (23-25).

For each study that had published results we extracted, whenever possible, the fully adjusted relative risk (RR) for invasive breast cancer and its 95% CI comparing night shift workers versus non-night shift workers (ever versus never night shift work where available or current versus not current night shift work) and for two categories of long duration night work (≥20 years, and ≥30 years) compared with never night workers. Summary relative risks combining study-specific results were estimated by calculating the weighted average of the study-specific logarithms of the relative risks, with weights proportional to the inverses of the variances of the study-specific log relative risks. (NB calculation of such a weighted average is sometimes referred to as a fixed-effect meta-analysis.) Chi-squared tests were used to assess heterogeneity across studies.

We identified 10 prospective studies, including the three current studies, with individual level data on night work in relation to the risk of breast cancer (2-4,26-29). All 10 studies provided information that allowed a comparison of the breast cancer incidence rate ratio in night shift workers versus that in non-night workers (nine studies with data on ever versus never night shift workers and one with data on current versus not current night shift workers) that could be combined to estimate overall risk in night shift workers versus non-night shift workers. Eight of these studies provided relative risks for long duration night shift workers compared with those who had never worked at night (**Supplementary Table 8**) (although for one study night shift work duration was based on information on current night shift work combined with job tenure for the current job (27)), with data being available from eight of these studies on risk in women working at night for approximately 20 years or longer compared with never night shift workers (3,4,27-29) and from four studies for approximately 30 years or longer of night shift work versus never night shift work (2,4,28).

For three of the studies that had only reported results for several categories of night shift workers compared with never night shift workers (2,3,28), to estimate the RR for ever versus never night workers we combined relative risks across the night shift work categories using a method for combining non-independent strata (30), which makes adjustment for the lack of independence of risks within each study (because the risks are all estimated with the same baseline). Using this method, for one study (28) we also combined relative risks for categories of long duration night shift work (>19.92-27.67 and >27.67 years) to estimate the relative risk in women working approximately 20 years or longer at night compared with never night workers. One further study did not report relative risks specifically for ≥20 years and so results from it are included for ≥30 years and for 15-29 years of night shift work (as in the 15-29 years category, most are likely to have worked night shifts for 20 years or longer) (2), and relative risks from these two categories were combined (30) to provide a single relative risk for long duration night work for inclusion in the meta-analysis of ~20 years of longer compared with never night work.

The identified studies were assessed for quality with respect to criteria including study design, exposure definition and assessment, adjustment for confounders and the potential for bias resulting from differential recall or participation. The primary aims of the meta-analysis were to investigate risk for breast cancer in ever versus never night workers, and in long duration night workers (≥20 years, and ≥30 years) versus women who had never worked at night, and, as described above, all ten included studies provided relative risks for ever versus never night workers, while 8 had data available on long duration versus never night work. Individual-level night shift exposure information was self-reported in 8 studies (the three studies reported here and 2-4,26,29) and obtained via linkage to a labour force survey in one study (27). In the tenth study it was obtained by combining individual-level information on employment in specific manufacturing processes within a particular factory (from factory personnel records for 80% of participants) with data on night shift work associated with each specific process in that factory (28). All the identified studies presented relative risks from a multivariable-adjusted statistical model that included established risk factors for breast cancer (with some additionally stating that the addition of other covariates had not materially altered findings), although the covariates included varied (see **Supplementary Table 8**). In order to provide reliable epidemiological evidence, with minimum methodological bias, on any relationship between night shift work and breast cancer incidence, only prospective studies were eligible for inclusion, and therefore, by design, the results from the studies included are not subject to bias introduced by differential recall or participation, with the exposure assessments having been conducted prior to diagnosis of breast cancer in 8 studies, and via independent sources in 2 studies (linkage to a labour force survey (27) and to individual-level work history information (28)).

*Format of results*

When several RRs are plotted, the RRs are represented by squares (with their 95% CIs as lines), each with area inversely proportional to the variance of the log RR, thereby indicating the amount of statistical information for that particular RR. Meta-analyses that combine information from more than one study involve inverse-variance weighted averages of the log RR values in the separate studies, yielding a result and its 95% CI that is plotted as a diamond.

|  |
| --- |

**SUPPLEMENTARY TABLES**

**Supplementary Table 1.** Baseline characteristics by reported night shift work duration in 519 062^†^ Million Women Study participants and results of follow-up for breast cancer

| **Baseline characteristics and follow-up for breast cancer** | | **Never night shifts**  **(n=450232)** | **Ever night shift work by night shift work duration** | | |
| --- | --- | --- | --- | --- | --- |
|  |  |  | **<10 years** | **10-19 years** | **≥20 years** |
|  |  |  | **(n=45922)** | **(n=13261)** | **(n=9647)** |
| **Baseline characteristics*** |  |  |  |  |  |
| Mean age at baseline, y (SD) | 68.8 (4.7) | 68.5 (4.6) | 68.2 (4.5) | 68.4 (4.5) |  |
| Socioeconomic status, % in lower third (N) | 32.5 (145392) | 35.9 (16355) | 37.7 (4995) | 37.0 (3546) |  |
| Working as a nurse for 10 years or longer, % (N) | 1.8 (7886) | 40.0 (18360) | 52.8 (7007) | 61.1 (5899) |  |
| Not married/living with a partner, % (N) | 17.8 (66529) | 22.5 (8610) | 25.0 (2683) | 26.3 (2039) |  |
| Nulliparous, % (N) | 11.0 (49313) | 10.0 (4589) | 10.8 (1432) | 14.4 (1387) |  |
| Mean number of children (parous women )(SD) | 2.3 (0.9) | 2.5 (1.0) | 2.5 (1.1) | 2.5 (1.1) |  |
| Mean age at first birth (parous women), y (SD) | 24.3 (4.2) | 23.9 (4.3) | 23.9 (4.6) | 24.0 (4.4) |  |
| Obese, % (N) | 14.7 (63554) | 18.0 (7941) | 21.1 (2691) | 22.6 (2089) |  |
| Strenuous physical activity, % > once a week (N) | 23.4 (103199) | 25.6 (11516) | 24.0 (3109) | 24.4 (2300) |  |
| Mean alcohol consumption, g/day (SD) | 6.4 (7.6) | 6.3 (7.7) | 5.9 (7.4) | 5.8 (7.5) |  |
| Current smoker, % (N) | 13.3 (57198) | 18.3 (8004) | 21.3 (2683) | 20.1 (1842) |  |
| First degree relative with breast cancer, % (N) | 9.7 (41339) | 10.2 (4371) | 10.0 (1231) | 9.4 (855) |  |
| Ever oral contraceptive user, % (N) | 63.0 (281855) | 68.1 (31112) | 67.3 (8861) | 64.7 (6198) |  |
| Ever menopausal hormone therapy user (women ≥55 years) | 54.2 (226674) | 62.3 (26898) | 62.2 (7669) | 60.8 (5476) |  |
| Mean hours of sleep (SD) | 6.8 (1.3) | 6.7 (2.0) | 6.6 (1.7) | 6.6 (1.5) |  |
| Take medication to sleep on most days, % (N) | 5.3 (226013) | 6.8 (3025) | 7.2 (916) | 6.9 (642) |  |
| More evening than morning type, % (N) | 28.8 (117848) | 31.9 (13273) | 34.2 (4094) | 38.5 (3315) |  |
| **Follow-up for breast cancer** | |  |  |  |  |
| Mean person-years of follow-up per woman | | 2.6 | 2.6 | 2.5 | 2.5 |
| Total number of incident breast cancers | | 4136 | 400 | 140 | 89 |

* Values are at recruitment, apart from living with a partner, which was recorded 3 years after recruitment, and age, working as a nurse, use of menopausal hormone therapy, sleep duration and chronotype, which were recorded at the same time as information on night shift work, which is the baseline for these analyses.

^†^ Duration of night shift work was unknown for 3184 night shift workers, including 44 cases.

**Supplementary Table 2.** Baseline characteristics by reported night shift work duration in 22 274^†^ women in EPIC-Oxford and results of follow-up for breast cancer

|  | | **Never night shifts** | **Ever night shift work by night shift work duration** | | |
| --- | --- | --- | --- | --- | --- |
|  | |  | **<10 years** | **10-20 years** | **≥20 years** |
| **Baseline characteristics and follow-up for breast cancer** | | **(n=19289)** | **(n=1819)** | **(n=705)** | **(n=461)** |
| **Baseline characteristics*** |  |  |  |  |  |
| Mean age at baseline, y (SD) | 58.0 (12.2) | 55.9 (12.0) | 54.7 (11.1) | 59.2 (10.1) |  |
| Socioeconomic status, % in lower third (N) | 33.0 (5628) | 36.5 (586) | 35.0 (214) | 30.4 (125) |  |
| Not living with a partner, % (N) | 29.6 (5693) | 33.2 (603) | 33.8 (238) | 34.9 (161) |  |
| Nulliparous, % (N) | 37.7 (7216) | 38.8 (700) | 42.6 (298) | 38.0 (173) |  |
| Mean number of children (parous women) (SD) | 2.2 (0.9) | 2.2 (1.0) | 2.2 (1.0) | 2.3 (0.9) |  |
| Mean age at first birth (parous women), y (SD) | 26.1 (4.7) | 26.0 (4.7) | 26.0 (4.8) | 26.2 (4.8) |  |
| Obese, % (N) | 5.6 (1053) | 7.0 (124) | 7.4 (51) | 10.9 (49) |  |
| Strenuous physical activity, % >2 hours/week (N) | 29.0 (5511) | 35.2 (637) | 35.4 (248) | 34.4 (156) |  |
| Mean alcohol consumption, g/day (SD) | 8.1 (9.8) | 8.0 (9.2) | 7.4 (8.6) | 7.7 (9.7) |  |
| Current smoker, % (N) | 8.5 (1638) | 11.1 (201) | 13.3 (93) | 10.4 (48) |  |
| Ever oral contraceptive user, % (N) | 77.2 (14 797) | 80.1 (1415) | 83.7 (589) | 79.9 (366) |  |
| Ever menopausal hormone user (women ≥55 years), % (N) | 40.2 (1420) | 47.3 (125) | 41.7 (30) | 47.9 (35) |  |
| Mean hours of sleep (SD) | 6.9 (1.1) | 6.8 (1.1) | 6.8 (1.2) | 6.7 (1.2) |  |
| Almost always take medication to sleep, % (N) | 1.9 (369) | 2.4 (44) | 2.4 (17) | 3.1 (14) |  |
| More evening than morning type, % (N) | 29.5 (5252) | 32.6 (552) | 36.4 (232) | 35.2 (148) |  |
| **Follow-up for breast cancer** | |  |  |  |  |
| Mean person-years of follow-up per woman | | 3.1 | 3.1 | 3.1 | 3.2 |
| Total number of incident breast cancers | | 153 | 15 | 11 | 1 |

^*^ Values are at recruitment, apart from age, sleep duration and chronotype, which were recorded at the same time as information on night shift work, which is the baseline for these analyses.

^†^ Duration of night shift work was unknown for 285 night shift workers, including 1 case.

**Supplementary Table 3.** Baseline characteristics by reported night shift work at recruitment in 251 045 women in UK Biobank and results of follow-up for breast cancer

|  | **Night shift work at recruitment** | | | |  |
| --- | --- | --- | --- | --- | --- |
|  | **Never/rarely** | **Sometimes** | **Usually** | **Always** |  |
| **Baseline characteristics and follow-up for breast cancer** | **(n=241972)** | **(n=5115)** | **(n=1338)** | **(n=2620)** |  |
| **Baseline characteristics*** | |  |  |  |  |
| Mean age at baseline, y (SD) | | 56.3 (8.0) | 50.8 (6.5) | 50.8 (6.5) | 51.7 (6.8) |
| Socioeconomic status, % in lower third (N) | | 32.9 (79450) | 44.5 (2270) | 44.6 (595) | 47.5 (1244) |
| Not living with a partner, % (N) | | 30.0 (72399) | 40.2 (2041) | 41.2 (549) | 41.6 (1086) |
| Nulliparous, % (N) | | 18.7 (45000) | 23.2 (1181) | 21.8 (291) | 17.3 (450) |
| Mean number of children (parous women )(SD) | | 2.2 (0.9) | 2.3 (1.0) | 2.3 (1.0) | 2.4 (1.0) |
| Mean age at first birth (parous women), y (SD) | | 26.0 (5.1) | 25.6 (5.4) | 25.4 (5.3) | 24.7 (5.3) |
| Obese, % (N) | | 23.6 (55962) | 28.2 (1415) | 30.2 (398) | 33.3 (857) |
| Vigorous physical activity, % > 2 hours/week (N) | | 17.5 (37364) | 22.0 (978) | 20.5 (240) | 22.4 (499) |
| Mean alcohol consumption, g/day (SD) | | 8.9 (10.9) | 9.2 (12.3) | 8.2 (11.0) | 7.3 (10.7) |
| Current smoker, % (N) | | 8.7 (20865) | 14.7 (751) | 14.6 (195) | 17.0 (444) |
| First degree relative with breast cancer, % (N) | | 6.8 (15522) | 5.4 (259) | 5.7 (72) | 5.7 (140) |
| Ever oral contraceptive user, % (N) | | 81.1 (195681) | 85.5 (4360) | 85.7 (1142) | 83.1 (2169) |
| Ever menopausal hormone user (women ≥55 years), % (N) | | 51.4 (70624) | 48.9 (703) | 51.1 (204) | 50.0 (454) |
| Mean hours of sleep in every 24 hours (SD) | | 7.2 (1.1) | 7.0 (1.1) | 7.0 (1.1) | 6.9 (1.3) |
| More evening than morning type, % (N) | | 36.4 (80068) | 41.7 (1922) | 46.9 (565) | 51.3 (1196) |
| **Follow-up for breast cancer** |  |  |  |  |  |
| Mean person-years of follow-up per woman | 3.8 | 3.8 | 3.8 | 3.9 |  |
| Total number of incident breast cancers | 2653 | 34 | 12 | 21 |  |

* Values are at recruitment, which is the baseline for these analyses.

**Supplementary Table 4.** Breast cancer incidence rate ratio* (RR) by night shift work and time since last worked night shifts reported at baseline in 518 185^†^ Million Women Study participants

|  | **Less than 10 years since last worked night shifts** | | | | |  | **10 or more years since last worked night shifts** | | | | | |
| --- | --- | --- | --- | --- | --- | --- | --- | --- | --- | --- | --- | --- |
|  | **Cases** | |  | **Multivariable-adjusted** | | |  | **Cases** | |  | **Multivariable-adjusted** | |
| **Night shift work** | **N** | **Mean years of night shift work (SD)** |  | **RR***^‡^ | | **95% CI**^‡^ |  | **N** | **Mean years of night shift work (SD)** |  | **RR***^‡^ | **95% CI**^‡^ |
| Ever worked at night |  |  |  |  | |  |  |  |  |  |  |  |
| Never | 4136 | - |  | 1.00 | |  |  | 4136 | - |  | 1.00 |  |
| Ever | 156 | 14.0 |  | 1.10 | | 0.94-1.30 |  | 474 | 7.0 |  | 0.96 | 0.87-1.06 |
| Years of night shift work |  |  |  |  |  |  |  |  |  |  |  |  |
| Never | 4136 | - |  | 1.00 | |  |  | 4136 | - |  | 1.00 |  |
| <10 | 55 | 3.8 |  | 0.97 | | 0.74-1.26 |  | 329 | 3.4 |  | 0.92 | 0.82-1.03 |
| 10 – 19 | 52 | 12.9 |  | 1.41 | | 1.07-1.86 |  | 83 | 12.3 |  | 1.01 | 0.81-1.26 |
| ≥ 20 | 42 | 28.1 |  | 0.98 | | 0.72-1.33 |  | 41 | 25.5 |  | 0.96 | 0.70-1.30 |
| Trend test |  |  |  | P = 0.42^‡^ | |  |  |  |  |  | P = 0.61^‡^ |  |

* Relative to never night workers, stratified by region, with attained age as the underlying time variable, and adjusted for socioeconomic status, parity and age at first birth, body mass index, alcohol intake, strenuous physical activity, family history of breast cancer, age at menarche, oral contraceptive use, smoking, living with a partner, and use of menopausal hormone therapy.

^†^ Time since last worked at night was unknown for 4061 night shift workers, including 43 cases.

^‡^ CI = confidence interval; RR = incidence rate ratio from Cox regression models; P is from a two-sided test for trend using mean night shift work duration within each duration category.

**Supplementary Table 5.** Breast cancer incidence rate ratio* (RR) by night shift work in 402 59 Million Women Study participants who had worked as a nurse for a total of ten years or longer

|  | **Cases** | |  | **Minimally-adjusted** | **Multivariable-adjusted** | |  |
| --- | --- | --- | --- | --- | --- | --- | --- |
| **Night shift work** | **N** | **Mean years of night shift work (SD)** |  | **RR***^‡^ | **RR**^†‡^ | **95% CI**^‡^ |  |
| Ever worked at night |  |  |  |  |  |  |  |
| Never | 80 | - |  | 1.00 | 1.00 |  |  |
| Ever | 319 | 9.9 |  | 0.98 | 0.96 | 0.75-1.23 |  |
| Years of night shift work | |  |  |  | | | |
| Never | 80 | - |  | 1.00 | 1.00 |  | |
| <10 | 180 | 3.9 |  | 0.96 | 0.95 | 0.73-1.24 | |
| 10 – 19 | 72 | 12.6 |  | 1.04 | 1.00 | 0.73-1.38 | |
| ≥ 20 | 53 | 27.2 |  | 0.92 | 0.88 | 0.62-1.25 | |
| Trend test |  |  |  |  | P = 0.60^‡^ |  | |

* Relative to never night workers, stratified by region and with attained age as the underlying time variable.

^†^ Relative to never night workers, stratified by region, with attained age as the underlying time variable, and adjusted for socioeconomic status, parity and age at first birth, body mass index, alcohol intake, strenuous physical activity, family history of breast cancer, age at menarche, oral contraceptive use, smoking, living with a partner, and use of menopausal hormone therapy.

^‡^ CI = confidence interval; RR = incidence rate ratio from Cox regression models; P is from a two-sided test for trend using mean night shift work duration within each duration category.

**Supplementary Table 6.** Breast cancer incidence rate ratio* (RR) by night shift work in 22 559 women in EPIC-Oxford

|  | **Cases** | |  | **Minimally-adjusted** | **Multivariable-adjusted** | | | |
| --- | --- | --- | --- | --- | --- | --- | --- | --- |
| **Night shift work** | **N** | **Mean years of night shift work** |  | **RR***^$^ | **RR**^†$^ | **95% CI**^$^ | | |
| Ever worked night shifts |  |  |  |  |  |  | | |
| Never | 153 | - |  | 1.00 | 1.00 |  | | |
| Ever | 28 | 9.4 |  | 1.07 | 1.07 | 0.71-1.62 | | |
| Years of night shift work^‡^ |  |  |  |  |  |  |  |  |
| Never | 153 | - |  | 1.00 | 1.00 |  | | |
| <10 | 15 | 4.8 |  | 1.12 | 1.18 | 0.69-2.01 | |  |
| 10 – 19 | 11 | 13.8 |  | 1.95 | 1.92 | 1.03-3.57 | |  |
| ≥ 20 | 1 | 30 |  | 0.24 | 0.22 | 0.03-1.61 | |  |
| Trend test |  |  |  |  | P = 0.75^$^ |  | | |

* Relative to never night workers, stratified by method of recruitment and region of residence and with attained age as the underlying time variable.

^†^ Relative to never night workers, stratified by method of recruitment and region of residence, with attained age as the underlying time variable, and adjusted for socioeconomic status (Townsend deprivation index), parity and age at first birth, body mass index, alcohol intake, strenuous physical activity, age at menarche, oral contraceptive use, smoking, married/living with a partner, and use of menopausal hormone therapy.

^‡^ Duration of night shift work was unknown for 285 night shift workers, including 1 case.

^$^ CI = confidence interval; RR = incidence rate ratio from Cox regression models; P is from a two-sided test for trend using mean night shift work duration within each duration category.

**Supplementary Table 7**. Breast cancer incidence rate ratio* (RR) by night shift work at recruitment in Million Women Study, EPIC-Oxford and UK Biobank women excluding women with missing data for one or more covariates^†^

|  | **Night shift work category** | **Cases** | | **Multivariable-adjusted** | | | | | | |
| --- | --- | --- | --- | --- | --- | --- | --- | --- | --- | --- |
| **Study** |  | **Number** |  | **RR***^‡^ | | | **95% CI***^‡^ | | | |
| **Million Women Study** | Ever worked at night |  |  |  | | |  | | | |
|  | Never | 2814 |  | 1.00 | | |  | | | |
|  | Ever | 443 |  | 1.00 | | | 0.91-1.11 | | | |
|  | Years of night shift work | |  |  |  | | |  |  |  |
|  | Never | 2814 |  | 1.00 | | |  | | |  |
|  | <10 | 270 |  | 0.93 | | | 0.82-1.06 | | |  |
|  | 10 – 19 | 91 |  | 1.17 | | | 0.95-1.44 | | |  |
|  | ≥ 20 | 56 |  | 0.98 | | | 0.76-1.28 | | |  |
|  | Trend test |  |  | | | P^‡^ = 0.72 | | |  | |
| **EPIC-Oxford** | Ever worked at night |  |  |  | | |  | | | |
|  | Never | 120 |  | 1.00 | | |  | | | |
|  | Ever | 20 |  | 1.11 | | | 0.70-1.77 | | | |
|  | Years of night shift work | |  |  |  | | |  |  |  |
|  | Never | 120 |  | 1.00 | | |  | | |  |
|  | <10 | 11 |  | 1.13 | | | 0.60-2.12 | | |  |
|  | 10 – 19 | 10 |  | 2.14 | | | 1.10-4.15 | | |  |
|  | ≥ 20 | 1 |  | 0.31 | | | 0.04-2.22 | | |  |
|  | Trend test |  |  | | | P^‡^ = 0.90 | | |  | |
| **UK Biobank** | Night shift work at recruitment |  |  |  | | |  | | | |
|  | Never/rarely | 2094 |  | 1.00 | | |  | | | |
|  | Yes, at least sometimes | 52 |  | 0.77 | | | 0.58-1.01 | | | |

* Stratified by region, with attained age as the underlying time variable, and adjusted for socioeconomic status, parity and age at first birth, body mass index, alcohol intake, strenuous physical activity, family history of breast cancer (except for EPIC-Oxford in which this information is not available), age at menarche, oral contraceptive use, smoking, married/living with a partner, and use of menopausal hormone therapy.

^†^ The characteristics that are included as covariates in the multivariable Cox regression model are socioeconomic status (Townsend deprivation index), parity and age at first birth, body mass index, alcohol intake, strenuous physical activity, family history of breast cancer (for the Million Women Study and UK Biobank), age at menarche, oral contraceptive use, smoking, married/living with a partner, and use of menopausal hormone therapy.

^‡^ CI = confidence interval; RR = incidence rate ratio from Cox regression models. P is from a two-sided test for trend using mean night shift work duration within each duration category.

**Supplementary Table 8.**  Characteristics of prospective studies and previous meta-analyses of night shift work history and duration in relation to breast cancer risk* ^†^

| **Prospective Cohort Studies** | |  |  |  |  |  |  |  |  |
| --- | --- | --- | --- | --- | --- | --- | --- | --- | --- |
| **Authors, Year (Reference)** | **Study (Age, follow-up)** | **Study population (Country)** | **Night shift work definition** | **Night shift work prevalence** | **Exposure categories*** | **Cases**  **Exposed/ unexposed** | **Relative risk**^†^ | **95% CI** | **Covariates** |
| Schernhammer et al., 2001 (2,31) | Nurses’ Health Study I  78562 women  2441 incident breast cancers  (Mean age at baseline: 54.3 y in never night workers, 60.4 y in ≥ 30 y night workers  Mean follow-up: 10 y)  *Updated results published in abstract form in 2014*  *5575 incident breast cancers*  *12 additional years of follow-up* | Registered nurses recruited in 1976 and surveyed for shift work history in 1988 (U.S.A.) | Rotating night shifts ≥ 3 nights per month in addition to days and evenings in that month | 59.6% ever night work | Ever vs never night work  ≥ 15-29 years night work vs never  ≥ 30 years night work vs never  *≥ 30 years night work vs never* | 1516/925  134/925  58/925  *Not given* | 1.09‡  1.08  1.36  *0.95* | 1.00-1.19  0.90-1.30  1.04-1.78  *0.77-1.17* | Age, age at menarche, parity and age at first birth combined, weight change between age 18 years and menopause, body mass index at 18, family history of breast cancer, benign breast disease, oral contraceptive use, current alcohol consumption, time period, age at menopause, use of postmenopausal hormones and menopausal status combined, and height |
| Schernhammer et al., 2006 (3,31) | Nurses’ Health Study II  115022 women  1352 incident invasive breast cancers  (Mean age at baseline: 40.5 y in never night workers, 45.0 y in ≥ 30 y night workers  Mean follow-up: 12 y)  *Updated results published in abstract form in 2014*  *2869 incident breast cancers* | Registered nurses recruited and first surveyed for shift work history in 1989 (U.S.A.) | Rotating night shifts ≥ 3 nights per month in addition to days and evenings in that month | 69.0% ever night work | Ever vs never night work  ≥ 20 years vs never night work  *≥ 20 years vs never night work*  *(Baseline ≥ 20 years vs never night work)* | 911/441  15/441  *Not given*  *Not given* | 0.98‡  1.79  *1.33*  *2.11* | 0.88-1.01  1.06-3.01  *0.93-1.89*  *1.21-3.66* | Age, age at menarche, menopausal status, age at menopause, age at first birth and parity combined, body mass index, current alcohol consumption, oral contraceptive use, use of postmenopausal hormones, smoking status, benign breast disease, family history of breast cancer, and physical activity |

| Pronk et al., 2010 (4) | Shanghai Women’s Health Study  73049 women  349 incident breast cancers  (Mean age at baseline: 52.5  Mean follow-up: 9 y) | General urban population  (Shanghai, China) | Self-reported: night shift work starting after 10pm ≥ 3 nights per month for at least 1 year | 26.2% ever night work | Ever vs never/ < 1 y night work  >17 years of night shift work vs no night work  >20 years of night shift work vs no night work  >30 years of night shift work vs none | 73/276  19/276  Not given/276  Not given/276 | 0.9  0.8  0.7  0.9 | 0.7-1.1  0.5-1.2  0.4-1.2  0.4-2.0 | Age, education, family history of breast cancer, number of pregnancies, age at first birth and occupational physical activity |
| --- | --- | --- | --- | --- | --- | --- | --- | --- | --- |
| Knutsson et al., 2013 (26) | W.O.L.F. (Work, Lipids, and Fibrinogen) Study  4036 women  94 incident breast cancers    (Age at baseline: 19-70y.  Mean follow-up: 12.4 y) | Longitudinal occupational cohort study of employees from 60 different public and private companies in several sectors, recruited 1992 to 1995, 1996 to 1997, and 2000 to 2003 (Sweden) | Responses indicating shift work with night work on at least 1 survey.  Reference group is day workers rather than shift workers without night work | 13.6% ever night work | Shifts with night work vs day work only  (No data available on long duration night work Estimated mean years of night shifts for night workers: 9.4 y) | 14/60 | 2.02 | 1.03-3.95 | Number of children and alcohol consumption. Attained age as the underlying time variable. (The following variables were not kept in the model because they only marginally influenced the HR: body mass index, waist-hip ratio, educational level, current smoking, ever smoker, menopausal status, and treatment with hormones other than oral contraceptives.) |
| Koppes et al., 2014 (27) | Dutch Labor Force Survey  285723 women  2531 with hospital admission for breast cancer  (Mean follow-up: 7 y) | Dutch labour force survey participants aged 15-64 who had non-missing data on night work. Dutch Labor Force Surveys of 1996 until 2009, were individually linked with National registers on hospital admission (Netherlands) | Participants with a paid job for 12 h per week or more who when asked “Do you work at nights, meaning between midnight and 6 am?” responded “Yes, regularly” (rather than “No” or “Yes, sometimes”) | 6.7% (ever regular night work) | Regular night work in current job and vs no night work  ≥ 20 years in current job and working nights regularly in current job vs no night work | 117/2312  Not given | 0.87  0.95 | 0.72-1.05  0.62-1.45 | Adjusted for frequency of current night work, age, region of origin, children in the household, education, occupation and contractual working hours |

| Li et al., 2015 (28) | Shanghai Textile Workers  Case-cohort study of 1709 incident breast cancer cases and 4780 non-cases  (from a prospective study of 267400 women)  (Age at baseline: 30-66 y  Mean follow-up: 5.2 and 10.9 y for cases and non-cases, respectively) | Shanghai textile workers (China) | Night work (continuous working 24:00 to 05:00 h) as part of a rotating shift pattern, based on factory level data on shift work by job type with linkage to each participant’s work history | 51.0%  (ever shift work in non-cases) | Ever vs never rotating night shift work  >19.92 years vs no night shift work  >27.67 years vs no night shift work | 1152/557  576/557  287/557 | 0.99‡  0.96‡  0.88 | 0.95-1.03  0.86-1.06  0.74-1.05 | Age at the beginning of follow-up  (Results not appreciably altered by further adjustment for number of live births, age at first live birth, and alcohol intake) |
| --- | --- | --- | --- | --- | --- | --- | --- | --- | --- |
| Äkerstedt et al., 2015 (29) | Swedish Twins  13656 women  463 incident breast cancers  (Mean age at baseline: 51.8 y for day workers, 51.1 y for night workers  Mean follow-up: 8.7 years) | Twins born before 1959, participating in the Screening Across the Lifespan Twin (SALT) study by the Swedish Twin Registry (STR), who were aged 41-60 y at interview and followed-up in the Swedish Cancer Registry (Sweden) | Night work for 1-45 y based on the question “For how many years have you had working hours that meant you worked at night at least now and then” | 24.9% ever night work | Night work for 1-45 y vs no night work  >20 years vs no night work | 109/354  18/354 | 0.94  1.68 | 0.73- 1.22  0.98-2.88 | Age, education level, tobacco consumption, body mass index, having children, coffee consumption, previous cancer, use of hormones including oral contraceptives |
| Travis et al.  [this report] | Million Women Study  522246 women  4809 incident breast cancers  (Mean age at baseline shift work survey: 68.7 y  Mean follow-up: 2.6 y) | Participants recruited 1996–2001 when aged 50–64 years via the NHS breast screening centres in England and Scotland, and who provided information on their history of night shift work in 2009-2012 and were followed up for cancer incidence and death (UK) | Regular work at night or on night shifts (at any time between midnight and 06:00 hours, for at least 3 nights per month) for at least 1 year | 13.8% ever night shift work | Ever vs never night shift work  ≥ 20 years vs never night shift work  ≥30 years vs never night shift work | 673/4136  89/4136  32/4136 | 1.00  1.00  0.98 | 0.92-1.08  0.81-1.23  0.69-1.39 | Stratified by region and adjusted for deprivation, parity and age at first birth, body mass index, alcohol intake, smoking, strenuous physical activity, family history of breast cancer, age at menarche, living with partner, use of oral contraceptives, use of postmenopausal hormone therapy |

| Travis et al.  [this report] | EPIC-Oxford  22559 women  181 incident breast cancers  (Mean age at baseline: 57.8 years  Mean follow-up: 3.12 y) | Participants recruited 1993–1999 when aged 20 years or older, and who provided information on their history of night shift work in 2010 and were followed up for cancer incidence and death (UK) | Regular work at night, on night shifts, or on call at night (for at least one night per month or 12 nights per year) for at least 1 year | 14.6% ever night shift work | Ever vs never night shifts  ≥ 20 years vs never night shifts | 28/153  1/153 | 1.07  0.22 | 0.71-1.62  0.03-1.61 | Stratified by region and method of recruitment (postal  or GPs) and adjusted for deprivation, parity and age at first birth, body mass index, alcohol intake, smoking,  strenuous physical activity, age at menarche, married or living with a partner, use of oral contraceptives, use of postmenopausal hormone therapy |
| --- | --- | --- | --- | --- | --- | --- | --- | --- | --- |
| Travis et al.  [this report] | UK Biobank  251045 women  2720 incident breast cancers  (Mean age at baseline: 56.1 years  Mean follow-up: 3.8 y) | Participants recruited 2006-2010 when aged 40-69 years, and who provided information on their current employment and were followed up for cancer incidence and death (UK) | Current job involves night shift work (sometimes, usually or always) | 3.6% current night shift work | Current vs not current night shift work  (Information not available on night shift work duration) | 67/2653 | 0.78 | 0.61-1.00 | Stratified by region and adjusted for deprivation , parity and age at first birth, body mass index, alcohol, smoking, vigorous physical activity, family history of breast cancer, age at menarche, living with a partner, use of oral contraceptives, use of postmenopausal hormone therapy |
| **Summary of previous meta-analyses** | |  |  |  |  |  |  |  |  |
| **Authors, Year (Reference)** | **Inclusion period (up to)** | **Study designs eligible** | **Number of shift work studies included** | **Main shift work exposure**  **(study included)** | | | **Relative risks** | **(95% CI)** |  |
| Megdal et al., 2005 (11) | January 2005 | Prospective  Retrospective | 2  4 | ‘Night work’ (all studies) | | | 1.51 | (1.36-1.68) ^§^ | |
| Ijaz et al., 2013 (12) | October 2012 | Prospective  Retrospective | 4  12 | Per 5-years of shift work (‘cohort studies’)  Per 5-years of shift work (‘case-control studies’) | | | 1.09 | (0.97-1.05)  (1.02-1.20) |  |
| Jia et al., 2013 (13) | September 2012 | Prospective  Retrospective | 4  9 | Ever night shift work (all studies)  Ever night shift work (‘cohort studies’\|\|)  Ever night shift work (‘case-control studies’)  ≥15 years night shifts (all studies) | | | 1.20  1.32  1.08  1.15 | (1.08-1.33)  (1.17-1.50) \|\|  (0.97-1.21)  (1.03-1.29) | |
| Kamdar et al., 2013 (14) | March 2012 | Prospective  Retrospective | 4  8 | Ever night shift work (all studies)  ≥8 years night shifts (all studies) | | | 1.21  1.04 | (1.00-1.47) ¶  (0.92-1.18) ¶ | |
| Wang et al., 2013 (15) | May 2013 | Prospective  Retrospective | 3  7 | Ever night shift work (all studies)  Per 5-years of nights (all studies)  Per 5-years of nights (prospective studies)  Per 5-years of nights (retrospective studies) | | | 1.19  1.03  1.02  1.06 | (1.05-1.35)  (1.01-1.05)  (1.00-1.04)  (1.02-1.09) |  |
| He et al., 2015 (16) | January 2014 | Prospective  Retrospective | 4  11 | ‘Shift work’ (all studies) | | | 1.19 | (1.08-1.32) |  |
| Lin et al., 2015 (17) | October 2014 | Prospective | 5 | ‘Shift work dose effect’ (unit not given)  >20-year exposure | | | 1.06  1.09 | (1.01-1.10)  (1.01-1.17) |  |

^*^ Reference: Women not exposed to night shift work.

^†^ Relative risks for a comparison of (1) night shift workers versus non-night workers (reference group), and (2) long duration night shift workers versus non-night workers (reference group)

^‡^ Relative risks were not available in the published manuscript and so were calculated by combining published relative risks for the relevant night shift work categories compared with never night shift workers.

^§^ The authors also provided a pooled relative risk estimate of 1.44 (1.26-1.65) for female airline cabin crew and an overall pooled estimate of 1.48 (1.36-1.61) for female night workers and female airline cabin crew combined.

|| Relative risks based on a meta-analysis of data from 4 prospective cohort studies and 1 nested case-control study of female radio and telegraph operators in which exposure ascertainment was classified by the authors of the meta-analysis as prospective, although the information on night shift work was ascertained from job histories on board ships collected after selection of cases and controls, with shift work bring classified by a shipping journalist and researcher with knowledge of the recent history of such ships, and shift-work being classed as frequent exposure to the radio room both day and night.

¶ The summary relative risk also includes the results from studies of work on overnight or international flights.


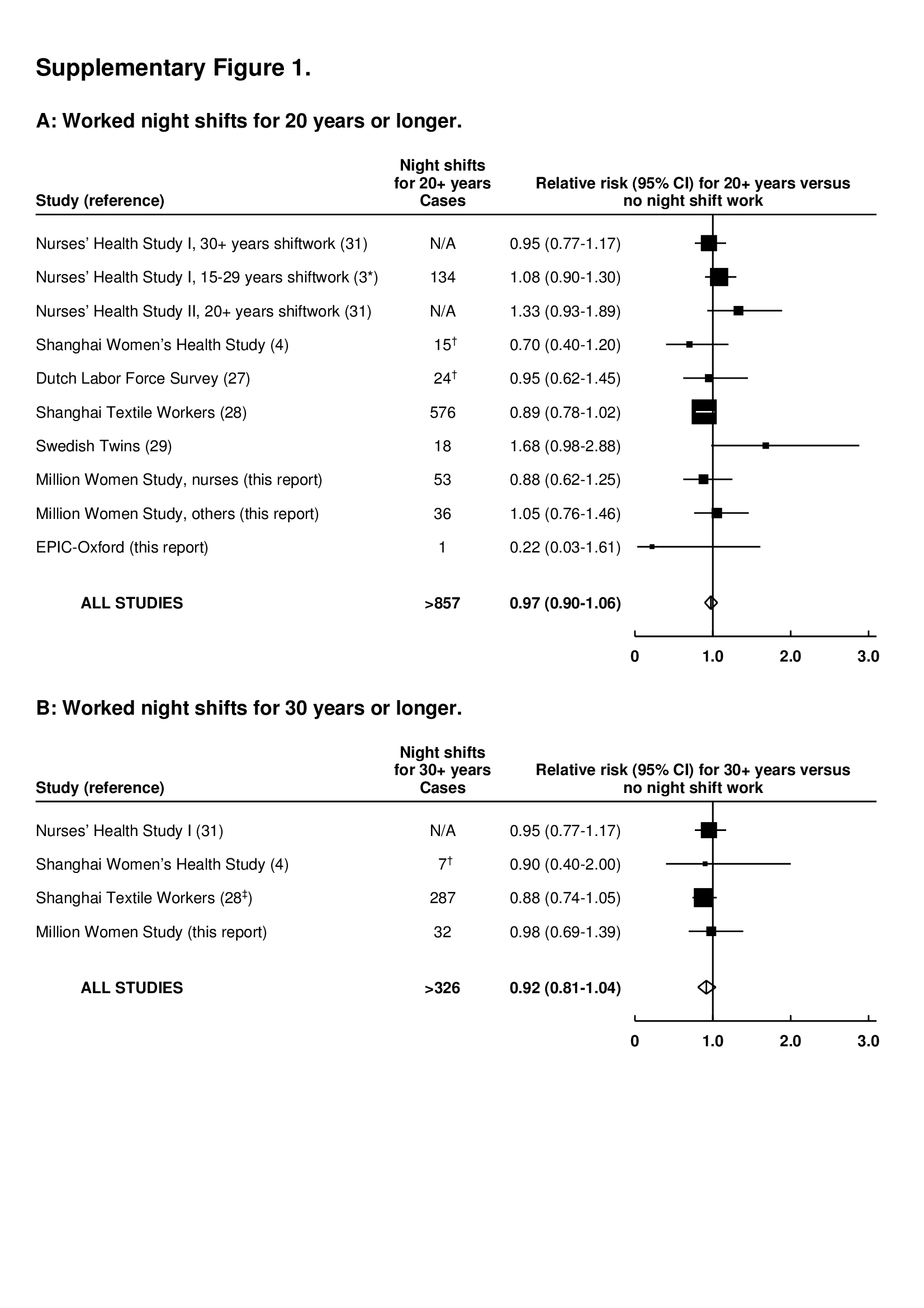


**Supplementary Figure 1.** Meta-analysis of prospective studies on the risk of breast cancer associated with long duration night shift work, with updated results from the Nurses’ Health Studies. **A)** Worked night shifts for 20 years or longer versus never worked night shifts. **B)** Worked night shifts for 30 years or longer versus never worked night shifts. * = Results for 20-29 years not reported separately. ^†^ = Approximate numbers, estimated from confidence limits. ^‡^ = Worked night shifts for more than 27.5 years. Study-specific relative risks (RRs) are represented by squares (with their 95% confidence intervals [CIs] as lines), each with area inversely proportional to the variance of the log RRs. RRs were combined using inverse-variance-weighted averages of the log RRs in the separate studies, yielding a result and its 95% CI that is plotted as a diamond. N/A = Not available.

**References**

1. Wang XS, Travis RC, Reeves G, et al. Characteristics of the Million Women Study participants who have and have not worked at night. *Scand J Work Environ Health.* 2012;38(6):590-599.

2. Schernhammer ES, Laden F, Speizer FE, et al. Rotating night shifts and risk of breast cancer in women participating in the Nurses' Health Study. *J Natl Cancer Inst.* 2001;93(20):1563-1568.

3. Schernhammer ES, Kroenke CH, Laden F, Hankinson SE. Night work and risk of breast cancer. *Epidemiology.* 2006;17(1):108-111.

4. Pronk A, Ji BT, Shu XO, et al. Night shift work and breast cancer risk in a cohort of Chinese women. *American Journal of Epidemiology.* 2010;171(9):953-959.

5. Cohen J. A coefficient of agreement for nominal scales. *Educational and Psychological Measurement.* 1960;20(1):37–46.

6. WHO. International statistical classification of diseases and related health problems, 10th revision. Geneva: World Health Organization, 1992.

7. Townsend P, Phillimore P, Beattie A. *Health and deprivation: inequality and the north.* London: Croom Helm 1988.

8. Davey GK, Spencer EA, Appleby PN, Allen NE, Knox KH, Key TJ. EPIC-Oxford: lifestyle characteristics and nutrient intakes in a cohort of 33 883 meat-eaters and 31 546 non meat-eaters in the UK. *Public Health Nutr.* 2003;6(3):259-269.

9. Collins R. What makes UK Biobank special? *Lancet.* 2012;379(9822):1173-1174.

10. Allen NE, Sudlow C, Peakman T, Collins R. UK biobank data: come and get it. *Sci Transl Med.* 2014;6(224):224ed4.

11. Megdal SP, Kroenke CH, Laden F, Pukkala E, Schernhammer ES. Night work and breast cancer risk: a systematic review and meta-analysis. *Eur J Cancer.* 2005;41(13):2023-2032.

12. Ijaz S, Verbeek J, Seidler A, et al. Night shift work and breast cancer - a systematic review and meta-analysis. *Scand J Work Environ Health.* 2013;39(5):431-447.

13. Jia Y, Lu Y, Wu K, et al. Does night work increase the risk of breast cancer? A systematic review and meta-analysis of epidemiological studies. *Cancer Epidemiol.* 2013;37(3):197-206.

14. Kamdar BB, Tergas AI, Mateen FJ, Bhayani NH, Oh J. Night shift work and risk of breast cancer: a systematic review and meta-analysis. *Breast Cancer Res Treat.* 2013;138(1):291-301.

15. Wang F, Yeung KL, Chan WC, et al. A meta-analysis on dose-response relationship between night shift work and the risk of breast cancer. *Ann Oncol.* 2013;24(11):2724-2732.

16. He C, Anand ST, Ebell MH, Vena JE, Robb SW. Circadian disrupting exposures and breast cancer risk: a meta-analysis. *Int Arch Occup Environ Health.* 2015;88(5):533-547.

17. Lin X, Chen W, Wei F, Ying M, Wei W, Xie X. Night shift work increases morbidity of breast cancer and all-cause mortality: a meta-analysis of 16 prospective cohort studies. *Sleep Med.* 2015;16(11):1381-1377.

18. Stroup DF, Berlin JA, Morton SC, et al. Meta-analysis of observational studies in epidemiology: a proposal for reporting. Meta-analysis Of Observational Studies in Epidemiology (MOOSE) group. *JAMA.* 2000;283(15):2008-2012.

19. Schwartzbaum J, Ahlbom A, Feychting M. Cohort study of cancer risk among male and female shift workers. *Scand J Work Environ Health.* 2007;33(5):336-343.

20. Hansen J. Increased breast cancer risk among women who work predominantly at night. *Epidemiology.* 2001;12(1):74-77.

21. Lie JA, Roessink J, Kjaerheim K. Breast cancer and night work among Norwegian nurses. *Cancer Causes Control.* 2006;17(1):39-44.

22. Tynes T, Hannevik M, Andersen A, Vistnes AI, Haldorsen T. Incidence of breast cancer in Norwegian female radio and telegraph operators. *Cancer Causes Control.* 1996;7(2):197-204.

23. Hansen J, Lassen CF. Nested case-control study of night shift work and breast cancer risk among women in the Danish military. *Occup Environ Med.* 2012;69(8):551-556.

24. Hansen J, Stevens RG. Case-control study of shift-work and breast cancer risk in Danish nurses: impact of shift systems. *Eur J Cancer.* 2012;48(11):1722-1729.

25. Lie JA, Kjuus H, Zienolddiny S, Haugen A, Stevens RG, Kjaerheim K. Night work and breast cancer risk among Norwegian nurses: assessment by different exposure metrics. *Am J Epidemiol.* 2011;173(11):1272-1279.

26. Knutsson A, Alfredsson L, Karlsson B, et al. Breast cancer among shift workers: results of the WOLF longitudinal cohort study. *Scand J Work Environ Health.* 2013;39(2):170-177.

27. Koppes LL, Geuskens GA, Pronk A, Vermeulen RC, de Vroome EM. Night work and breast cancer risk in a general population prospective cohort study in The Netherlands. *Eur J Epidemiol.* 2014;29(8):577-584.

28. Li W, Ray RM, Thomas DB, et al. Shift work and breast cancer among women textile workers in Shanghai, China. *Cancer Causes Control.* 2015;26(1):143-150.

29. Akerstedt T, Knutsson A, Narusyte J, Svedberg P, Kecklund G, Alexanderson K. Night work and breast cancer in women: a Swedish cohort study. *BMJ Open.* 2015;5(4):e008127.

30. Berrington A, Cox DR. Generalized least squares for the synthesis of correlated information. *Biostatistics.* 2003;4(3):423-431.

31. Schernhammer E. Nightshift work and breast cancer risk - good news, bad news? *Occup Environ Med* 2014;71 (Suppl. 1)(A121).
